# Genetic population dynamics of the critically endangered scalloped hammerhead shark (*Sphyrna lewini*) in the Eastern Tropical Pacific

**DOI:** 10.1002/ece3.9642

**Published:** 2022-12-28

**Authors:** Sydney P. Harned, Andrea M. Bernard, Pelayo Salinas‐de‐León, Marissa R. Mehlrose, Jenifer Suarez, Yolani Robles, Sandra Bessudo, Felipe Ladino, Andrés López Garo, Ilena Zanella, Kevin A. Feldheim, Mahmood S. Shivji

**Affiliations:** ^1^ Save Our Seas Foundation Shark Research Center and Guy Harvey Research Institute Nova Southeastern University Dania Beach Florida USA; ^2^ Charles Darwin Research Station Charles Darwin Foundation Galápagos Islands Ecuador; ^3^ Direccion Parque Nacional Galápagos Departamento de Ecosistemas Marinos Islas Galápagos Ecuador; ^4^ Universidad de Panamá, Centro Regional Universitario de Veraguas San Martín de Porres Panama; ^5^ Fundacion Malpelo y Otros Ecosistemas Marinos Bogotá Colombia; ^6^ Asociación Conservacionista Misión Tiburon, Playas del Coco Carrillo Guanacaste Costa Rica; ^7^ Pritzker Laboratory for Molecular Systematics and Evolution Field Museum of Natural History Chicago Illinois USA

**Keywords:** aggregations, conservation, Galápagos, genetic diversity, philopatry

## Abstract

The scalloped hammerhead shark, *Sphyrna lewini*, is a Critically Endangered, migratory species known for its tendency to form iconic and visually spectacular large aggregations. Herein, we investigated the population genetic dynamics of the scalloped hammerhead across much of its distribution in the Eastern Tropical Pacific (ETP), ranging from Costa Rica to Ecuador, focusing on young‐of‐year animals from putative coastal nursery areas and adult females from seasonal aggregations that form in the northern Galápagos Islands. Nuclear microsatellites and partial mitochondrial control region sequences showed little evidence of population structure suggesting that scalloped hammerheads in this ETP region comprise a single genetic stock. Galápagos aggregations of adults were not comprised of related individuals, suggesting that kinship does not play a role in the formation of the repeated, annual gatherings at these remote offshore locations. Despite high levels of fisheries exploitation of this species in the ETP, the adult scalloped hammerheads here showed greater genetic diversity compared with adult conspecifics from other parts of the species' global distribution. A phylogeographic analysis of available, globally sourced, mitochondrial control region sequence data (*n* = 1818 sequences) revealed that scalloped hammerheads comprise three distinct matrilines corresponding to the three major world ocean basins, highlighting the need for conservation of these evolutionarily unique lineages. This study provides the first view of the genetic properties of a scalloped hammerhead aggregation, and the largest sample size‐based investigation of population structure and phylogeography of this species in the ETP to date.

## Graphical abstract

The scalloped hammerhead shark is a Critically Endangered (IUCN Red List), circumglobally distributed species in warm temperate and tropical seas. This species exhibits both periods of restricted movements as well a strong migratory propensity and is known to form iconic, large seasonal aggregations at offshore oceanic islands. In this manuscript, we report on the population genetic dynamics of scalloped hammerheads within the Eastern Tropical Pacific (ETP—a region experiencing high levels of illegal, unreported and unregulated fishing), and the mitochondrial phylogeography of this species worldwide.

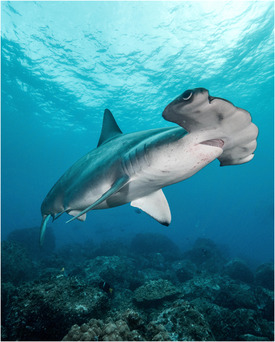

## 1 INTRODUCTION

Populations of many oceanic shark and ray species have declined dramatically since the onset of industrial fishing, with three‐quarters of these large‐bodied species facing increased risk of extinction, mainly from over‐exploitation (Pacoureau et al., 2021). Knowledge of the population dynamics of these oceanic sharks is required for guiding urgently needed, science‐based conservation management efforts, and understanding the ecology and evolutionary biology of these high trophic‐level marine predators.

Delineating management units (sensu Moritz, 1994) of oceanic shark species is made complex by their high vagility (Musick et al., 2004) and can be informed by an understanding of population genetic connectivity, including the influencing roles of dispersal and philopatry. Regional philopatry, as defined by Chapman et al. (2015), describes a highly mobile, roaming individual that typically returns to the region of its birth to reproduce, thereby limiting and/or restricting gene flow to within a much smaller geographic area than would otherwise be expected based on the vagility of the species alone. Nevertheless, given the migratory propensity of oceanic sharks, genetic differences among discrete reproductive units may be obscured by sampling and testing for differentiation among adults during non‐reproductive periods or by pooling samples across age classes, as varying life‐stages possess varying dispersal tendencies (Klein et al., 2019; McClain et al., 2022; Phillips et al., 2021). For instance, following parturition, many young‐of‐year (YOY) or juvenile sharks remain within  coastal nursery habitats for many months, as these habitats may serve as protection from predators or may be a location where there is an abundance of prey, whereas, in contrast, older life stages may disperse for feeding, reproduction, and other social behaviors. Thus, sampling highly vagile species of elasmobranchs at YOY stages or females undergoing parturition will likely provide the most useful information concerning genetic connectivity and how best to identify genetic management units.

**FIGURE 1:**
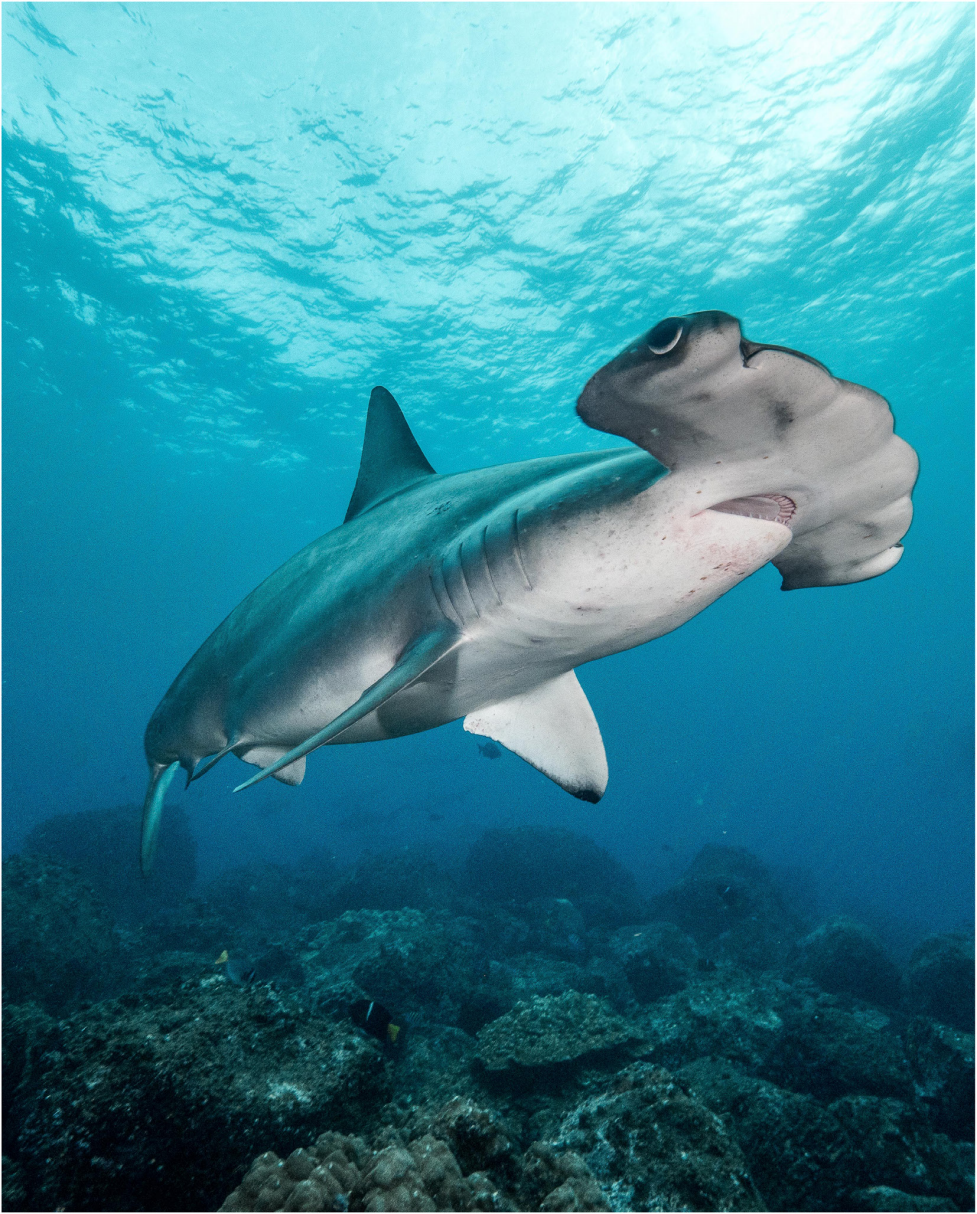
Scalloped hammerhead shark, *Sphyrna lewini*, in the Galápagos. Copyright: Pelayo Salinas de León.

An oceanic shark that has undergone steep declines globally is the scalloped hammerhead (*Sphyrna lewini*) (Rigby et al., 2019; Figure 1). This species occurs circumglobally in warm temperate to tropical seas, occupying habitats spanning near‐shore to pelagic environments. Nursery grounds for scalloped hammerheads occur mainly in very shallow coastal areas, coastal bays, and estuaries, where individuals may reside for upwards of a year (Duncan & Holland, 2006; Gallagher & Klimley, 2018). This hammerhead is migratory, with individuals undertaking long distance movements between distant habitats (>1000 km; Bessudo et al., 2011; Hoyos‐Padilla et al., 2014; Kohler & Turner, 2019; P. Salinas de León and M. Shivji, unpublished), indicating an innate capacity for widespread dispersal, albeit some individuals also show relatively restricted movements or resident behavior (Aldana‐Moreno et al., 2020; Wells et al., 2018). Some population genetic surveys have suggested that scalloped hammerhead females may be regionally philopatric (Chapman et al., 2009; Daly‐Engel et al., 2012; Pinhal et al., 2020; Rangel‐Morales et al., 2022). Scalloped hammerheads are also noted for forming seasonal aggregations at offshore oceanic islands and seamounts in some parts of its distribution, possibly to facilitate social interactions, utilize cleaning stations, mate, and/or use as a staging location from which to conduct nocturnal foraging excursions into the surrounding pelagic environment (Bessudo et al., 2011; Brown et al., 2016; Hearn et al., 2010; Klimley & Nelson, 1984; Salinas‐de‐León et al., 2017).

The Critically Endangered status (IUCN Red List; Rigby et al., 2019) of the scalloped hammerhead globally has resulted in several broad‐scale conservation policy measures (e.g., Appendix II listings on the Convention on International Trade in Endangered Species (CITES) and Convention on the Conservation of Migratory Species of Wild Animals), but this species is still harvested and traded for its meat and fins legally and illegally worldwide (Abercrombie et al., 2005; Rigby et al., 2019). The exploitation of scalloped hammerheads is particularly problematic in the Eastern Tropical Pacific (ETP), a highly biodiverse biogeographic region ranging from southern Mexico to northern Peru, including the Galápagos archipelago, where illegal, unreported and unregulated (IUU) fishing is widespread (Alava & Paladines, 2017; Enright et al., 2021; Espinoza et al., 2018).

Within the ETP, scalloped hammerheads form aggregations at offshore islands, including the northern Galápagos Islands of Darwin and Wolf, Malpelo Island (Colombia) and Cocos Island (Costa Rica) (Bessudo et al., 2011; Hearn et al., 2010; Nalesso et al., 2019), all designated as World Heritage Sites (UNESCO, 2021). The seasonal aggregations that form in the Galápagos Islands number in the few thousands and are composed mainly of adult females, many of whom are thought to be pregnant based on their expanded girth (Hearn et al., 2010; Ketchum et al., 2014; Salinas‐de‐León et al., 2016, 2017). To preserve this aggregation and other species in the waters of the Galápagos, the Ecuadorian government established the Galápagos Marine Reserve (GMR) and prohibited all shark fishing and landing within the GMR (Carr et al., 2013). However, scalloped hammerheads in the Galápagos are migratory, moving outside the bounds of the GMR into international waters where they face intense pressure from IUU fisheries (Alava et al., 2017; Carr et al., 2013; Dulvy et al., 2008). Notably, a temporal decline in abundance of females at the Galápagos aggregation site coincides with the appearance of YOY sharks in ETP mainland coastal nursery habitats (Nalesso et al., 2019) and recent telemetry work has documented adult female dispersal linking these two regions (P. Salinas de León and M. Shivji, unpublished). Furthermore, a direct parent‐offspring genetic connection between female scalloped hammerheads at Malpelo Island and YOY sharks in coastal nursery sites in Colombia was found (Quintanilla et al., 2015). These observations support the hypothesis that females in the Galápagos aggregations are using mainland coastal sites for parturition.

Understanding the population genetic dynamics and genetic diversity of scalloped hammerhead aggregations, and of this critically endangered shark across its ETP distribution, can provide insight into its biology, genetic health and resilience, and is of conservation management relevance (Hoban et al., 2021; Thomson et al., 2021). Eight previous studies examining the population genetic structure and phylogeography of scalloped hammerheads, from local to global scales, have included samples from at least one location in the broader eastern Pacific (Castillo‐Olguín et al., 2012; Daly‐Engel et al., 2012; Duncan et al., 2006; Green et al., 2022; Nance et al., 2011; Quintanilla et al., 2015; Rangel‐Morales et al., 2022; Villate‐Moreno et al., 2022). These studies have added to the body of knowledge about this species in this region, but their inferences have been constrained by either samples obtained from only one or a few sites, small samples sizes collected opportunistically from fisheries landings (given difficulties of sampling threatened megafauna), and in some cases sample sets pooled across variable demographic groups (YOY, juveniles, adults), factors which can lead to erroneous conclusions about population structure and dispersal patterns (McClain et al., 2022; Phillips et al., 2021). This previous work has offered inconsistent evidence of female philopatry in scalloped hammerheads within the ETP (e.g., Castillo‐Olguín et al., 2012; Daly‐Engel et al., 2012; Duncan et al., 2006; Nance et al., 2011; Rangel‐Morales et al., 2022), despite this behavior having been suggested elsewhere globally (Chapman et al., 2009; Daly‐Engel et al., 2012; Pinhal et al., 2020). Nevertheless, most of these previous studies indicate that gene flow throughout the ETP is at least somewhat restricted; however, at what spatial scale and whether this genetic differentiation is driven by female philopatry across the region remains unclear, thus warranting further investigation.

To add to existing information on scalloped hammerheads in the ETP, here we address four objectives. We: (1) assess the genetic diversity specifically of adult scalloped hammerheads from the Galápagos aggregations and adult sharks sampled from two other global regions to compare their genetic health and potential resiliency; (2) test for the presence of regional philopatry by ETP scalloped hammerheads by analyzing for population structure in YOY sharks collected from nursery sites and for relatedness in YOY within and among nursery sites; (3) analyze genetic kinship to determine whether (a) relatedness may be driving aggregation behavior in Galápagos adults and (b) the Galápagos aggregation adult females are using mainland coastal nursery sites for parturition; and (4) investigate matrilineal phylogeographic relationships of ETP scalloped hammerheads in the context of other global matrilines by combining our data with sequence data available from published studies.

## 2 METHODS

### 2.1 Sample collection and DNA extraction

**FIGURE 2:**
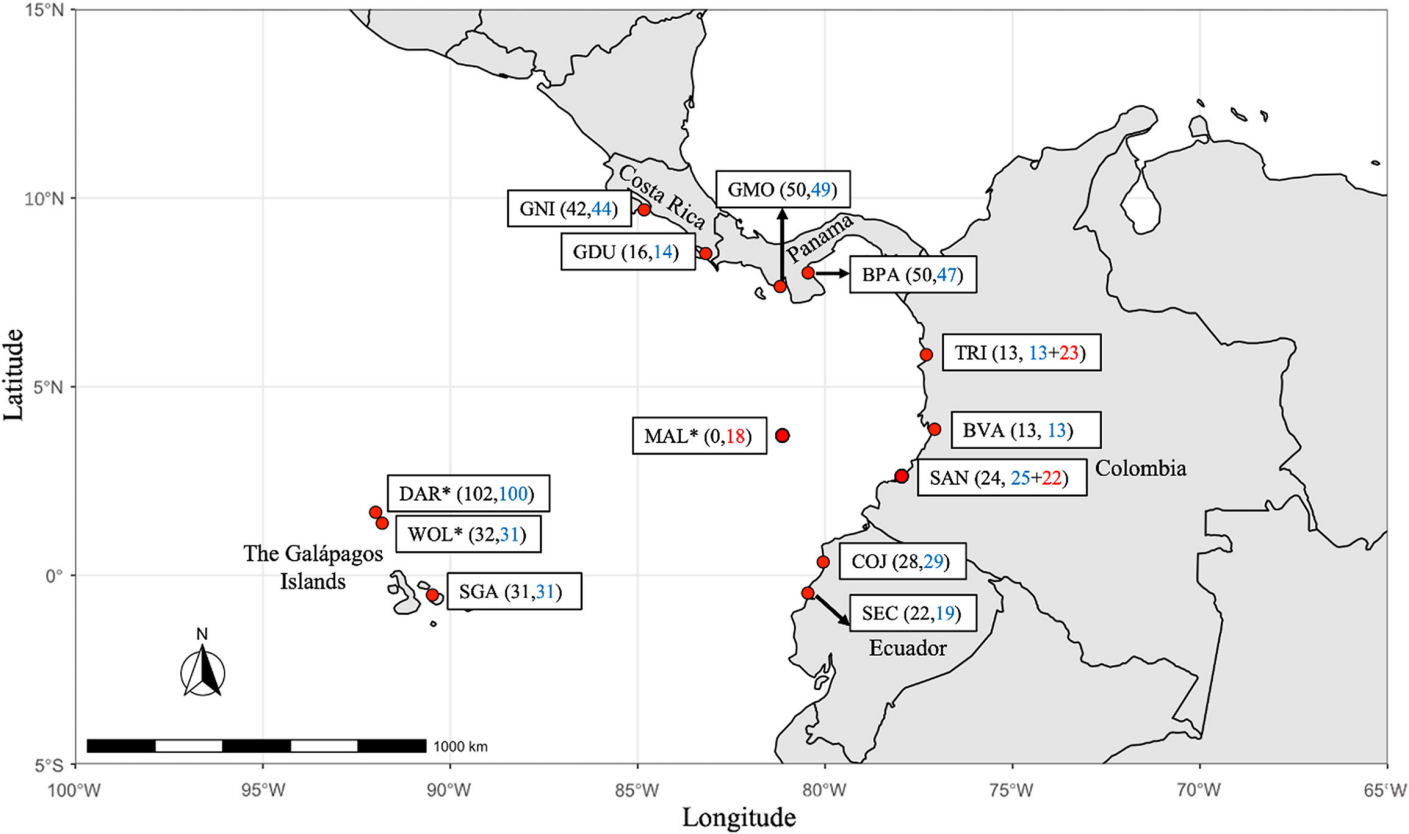
Sampling sites (13 red circles) and sample sizes (in brackets next to circles) of *Sphyrna lewini* in the Eastern Tropical Pacific. Black numbers in brackets represent the number of shark samples microsatellite genotyped at each location. Blue numbers in brackets represent the number of mitochondrial control region sequences from each location obtained in this study. Red numbers in brackets represent the number of mitochondrial control region sequences obtained from Quintanilla et al. (2015) and pooled with sequences from this study for analyses. Asterisks indicate locations of adult female samples. All other location samples are YOY (young‐of‐year) animals. Subpopulation abbreviations: BPA, Bahía Parita; BVA, Buenaventura; DAR, Darwin Arch; COJ, Cojimies; GNI, Golfo Nicoya; GDU, Golfo Dulce; GMO, Golfo de Montijo; TRI, Tribugá; MAL, Malpelo Island; SAN, Sanquianga; SEC, South Ecuador; SGA, South Galápagos; WOL, Wolf Island.

Scalloped hammerhead tissues were collected as tissue biopsies from individuals sampled from 14 globally distributed locations (hereafter referred to as subpopulations). Twelve of these subpopulations were sampled within the ETP (collection locations and final sample sizes of genotyped and sequenced sharks shown in Figure 2): samples from adult female sharks aggregating at two island sites within the Northern Galápagos [GAL: Wolf Island (WOL) and Darwin Arch (DAR)] were collected by free‐diving researchers using biopsy darts. Samples were also obtained from YOY sharks [Total Length (TL) <90 cm; Duncan & Holland, 2006] from nine ETP mainland coastal putative nurseries and/or artisanal fish markets. Scalloped hammerhead YOY fin samples were also collected from a South Galápagos (SGA) nursery area from sharks captured non‐lethally by gillnet. In addition to the 12 ETP sampling locations, fin tissues from non‐aggregating, putatively adult scalloped hammerheads (based on TL >150 cm; Compagno, 1984) were collected from two other distant locations: the western Indian Ocean during surveys of artisanal markets in the Seychelles (SEY) and the western North Atlantic [Florida, USA east coast; (FLA)] by sampling recreational fishery catches (Table 1). Samples from only adult sharks (GAL, SEY and FLA locations) were used in some of the subsequently described analyses to allow genetic comparison across equivalent age demographic groups.

**TABLE 1:** Sample sizes analyzed and genetic diversity indices for nine microsatellite DNA markers and 548‐bp sequence of the mitochondrial control region for *Sphyrna lewini* from global (adults only) and Eastern Tropical Pacific (ETP) (adults and YOY) subpopulations.

	Microsatellite DNA						Mitochondrial control region				
	*N*	*A*	*A*_R_	*H*_O_	*H*_E_	*F*_IS_	*N*	H	S	*h*	*π*
Global adults											
GAL (DAR + WOL)	134	16.2	12.0	0.759	0.759	0.004	131	8	7	0.550	0.0012
FLA	58	10.6	9.5	0.597	0.612	0.034	50	3	4	0.340	0.0010
SEY	68	14.6	12.7	0.758	0.796	0.057	50	2	2	0.184	0.0007
ETP only—All Groups											
DAR	102	15.4	7.0	0.757	0.759	0.057	100	8	7	0.561	0.0012
WOL	32	10.2	6.9	0.769	0.745	0.035	31	4	2	0.514	0.0011
SGA	31	10.8	7.2	0.728	0.749	0.051	31	1	0	0.000	0.0000
MAL	–	–	–	–	–	–	18^a^	3	2	0.582	0.0016
GNI	42	11.7	6.9	0.785	0.762	0.012	44	4	3	0.547	0.0012
GDU	16	8.9	7.1	0.702	0.739	0.134	14	4	3	0.659	0.0015
GMO	50	12.4	7.2	0.784	0.773	0.035	49	4	3	0.515	0.0010
BPA	50	12.1	7.2	0.796	0.768	0.012	47	5	3	0.550	0.0011
TRI	13	7.2	6.6	0.768	0.730	0.040	36^a^	4	3	0.491	0.0010
BVA	13	7.0	6.6	0.752	0.739	0.071	13	2	1	0.513	0.0009
SAN	24	10.2	7.2	0.804	0.756	0.002	47^a^	7	6	0.578	0.0013
COJ	28	10.1	6.9	0.756	0.751	0.012	29	4	3	0.650	0.0015
SEC	22	9.9	7.2	0.766	0.732	0.010	19	2	1	0.351	0.0006

Subpopulation abbreviations: BPA, Bahía Parita; BVA, Buenaventura; COJ, Cojimies; DAR, Darwin Arch; FLA, Florida; GAL, Galápagos adults pooled; GDU, Golfo Dulce; GMO, Golfo de Montijo; GNI, Golfo Nicoya; MAL, Malpelo Island; SAN, Sanquianga; SEC, South Ecuador; SEY, Seychelles; SGA, South Galápagos; TRI, Utria‐Tribugá; WOL, Wolf Island.

Microsatellite DNA: *n*, number of individuals included in analysis; *A*, number of alleles; *A*_R_, allelic richness; *H*_O_, observed heterozygosity; *H*_E_, expected heterozygosity. Mitochondrial Control Region: *n*, number of individuals included in analysis; H, number of haplotypes; S, number of segregating sites; *h*, haplotype diversity; *π*, nucleotide diversity.

^a^ Mitochondrial sample size includes samples from Quintanilla et al. (2015).

All tissue samples were stored in 95% undenatured ethanol. Genomic DNA was extracted using Qiagen DNeasy Blood and Tissue Kits according to the manufacturer's instructions (Qiagen Inc.).

### 2.2 Microsatellite marker amplification, genotyping, quality filtering, and summary statistics

Samples were genotyped at 10 microsatellite loci, including six loci from *Sphyrna lewini* (SLE018, SLE027, SLE033, SLE045, SLE038, SLE089) previously described by Nance et al. (2009), and four loci isolated in other shark species that also cross‐amplified in *S. lewini* [Cli‐12 from *Carcharhinus limbatus* (Keeney & Heist, 2003) and SMO3, SMO7, and SMO8 from *Sphyrna mokarran* (Feldheim et al., 2020)]. An additional nine loci from Nance et al. (2009) (identified below) were assessed for this study; however, initial genotyping showed these loci were either: (1) monomorphic across a subsample of genotyped individuals (i.e., SLE086), (2) difficult to consistently and robustly score/size due to the imperfect nature of the repeat pattern (i.e., SLE013, SLE025, SLE028, SLE053, SLE071, SLE077, and SLE081), or (3) on Sanger sequencing of microsatellite amplicons of the same length (bp), homoplasy of allele variants was identified (i.e., SLE054; single nucleotide polymorphisms were detected between two microsatellite alleles of the same fragment length). Each locus was amplified in a 12.5 μl polymerase chain reaction (PCR) with the following reagents: 1–2 μl DNA ranging from 0.5–20 ng/μl, 2 μl of dNTP mix containing 1.25 mM of each dNTP, 0.5 U HotStar *Taq*™ DNA polymerase, 1.25 μl HotStar *Taq*™ 10× reaction buffer (15 mM MgCl_2_), 0.165–0.25 μl MgCl_2_, 0.2–0.25 μl of 10 nM Forward primer with a 5′‐M13 tail, 0.2–0.5 μl of 10 nM Reverse primer, and 0.1–0.4 μl of 10 nM fluorescently labeled universal M13 primer (Schuelke, 2000). PCR reactions were carried out on an Applied Biosystems BioRad™ Thermal Cycler with the following thermal profile: 95°C for 15 min, 35 cycles of 94°C for 1 min, 1 min at the primer‐specific annealing temperature (i.e., TA = 50°C for Cli‐12, TA = 56°C for SMO7, TA = 60°C for SLE045, SLE089, SMO3, SMO8, and TA = 65°C for SLE018, SLE027, SLE033, SLE038), and 72°C for 2 min, followed by a final extension of 72°C for 20 min. Electrophoresis of amplified microsatellite loci was performed on an Applied Biosystems 3130 Genetic Analyzer. Alleles were sized using GeneScan LIZ 600 size standard and scored using the software GeneMapper v.3.7 (Applied Biosystems Inc.). Electropherograms were visually inspected by two researchers, and samples genotyped at fewer than seven microsatellite loci were discarded.

To ensure that no sample duplicates (e.g., repeated sampling of the same adults or YOYs) were included in downstream analyses, match analysis was performed as implemented in the Excel Microsatellite Toolkit (Park, 2001). Pairs of individuals possessing two or less mismatched alleles were considered likely duplicates (two mismatched alleles were allowed to account for possible genotyping error), and where matches were found, one multi‐locus genotype (along with its corresponding haplotype if present within the mitochondrial control region dataset) per putative duplicate pair was discarded. We tested for genotyping errors, null alleles, large‐allele dropout and stutter using Microchecker 2.2.3 (Van Oosterhout et al., 2004), and used FreeNa (Chapuis & Estoup, 2007) to directly estimate the frequency of null alleles with 1000 iterations. All loci were checked for subpopulation‐level deviations from Hardy–Weinberg Equilibrium (HWE) and linkage disequilibrium (LD) using GENEPOP on the Web (Rousset, 2008), with significance of deviations estimated using the Markov chain method and estimated probabilities corrected for multiple comparisons in R (p.adjust; R Core Team, 2020) with the false discovery rate (FDR) (Benjamini & Hochberg, 1995). Problematic loci, i.e., those with high levels of null alleles and/or deviations from HWE within more than three subpopulations, were excluded from downstream analysis. The statistical power of the suite of microsatellites given different levels of *F*_ST_ was estimated using POWSIM 4.1 (Ryman & Palm, 2006), assuming an effective population size (*N*_e_) of 500. Final POWSIM estimates were derived from 100 simulations per run, and Fisher's exact test analyses implemented 1000 dememorizations, 100 batches, and 1000 iterations per batch.

### 2.3 Microsatellite DNA: Analysis of population genetic structure within the ETP and among global adults

Microsatellite summary statistics [number of alleles (*A*), allelic richness (*A*_R_), inbreeding coefficient (*F*_IS_)] were determined for each locus and subpopulation using the program FSTAT 2.9.4 (1000 iterations, Goudet, 2001), while expected and observed heterozygosities (*H*_E_ and *H*_O_; Nei, 1978) were estimated with GenAlEx 6.5 (Peakall & Smouse, 2012).

To test for nuclear genetic population structure among scalloped hammerhead subpopulations in the ETP and among the globally sampled adults, we adopted both pairwise and cluster‐based analyses. First, pairwise nuclear genetic differentiation was assessed among: (1) adult shark subpopulations (SEY, FLA, and GAL) and (2) all ETP subpopulations (DAR, WOL and all YOY subpopulations). Pairwise metrics *F*_ST_ (Weir & Cockerham, 1984), standardized $G_{\mathrm{ST}}^{\prime \prime }$ (Meirmans & Hedrick, 2011), and Jost's *D*_EST_ (Jost, 2008) were estimated for all comparisons using GenAlEx; significance of values was determined using 999 permutations and estimated probabilities were adjusted with the FDR. Second, to test for isolation‐by‐distance (IBD; Bohonak, 2002) among ETP mainland putative nursery YOY subpopulations, a Mantel Test was performed in GenAlEx. Geographic distances were estimated between geographic coordinates using GenAlEx and significance of the correlation determined with 999 permutations. And third, nuclear microsatellite differentiation was further investigated using adegenet 2.1.5 (Jombart, 2008) by means of Discriminant Analysis of Principal Components (DAPC) (Jombart et al., 2010). Two DAPCs were performed: (1) global adult shark subpopulations only (i.e., SEY, FLA, and GAL) and (2) all ETP microsatellite genotypes (i.e., DAR, WOL subpopulations and all YOY nurseries). Clusters were pre‐assigned based on a priori subpopulations and all discriminant functions (DAs) were retained. The optimal number of principal components to include for each DAPC was determined using α‐score validation. DAPC outcomes were visualized as a scatterplot of genetic distance between groups.

### 2.4 Microsatellite DNA: Assessment of relatedness—Galápagos aggregation adults and ETP Young of Year sharks

We tested for relatedness and familial relationships in ETP scalloped hammerheads using a multi‐tiered approach. Potential parent‐offspring relationships among adult female sharks from the Galápagos aggregations and YOY sharks sampled from ETP nurseries were assessed using the programs Cervus 3.0.7 (Marshall et al., 1998) and Colony 2.0 (Jones & Wang, 2010). Cervus implements a pairwise likelihood‐based approach to assign offspring to the most likely true parent from a pool of candidate parents. Cervus was run three times with the parameter “proportion of candidate parents sampled” set to 0.1, 0.01, and 0.001.

Parentage and sibling relationships (full‐ and half‐siblings) among ETP sharks were assessed using Colony 2.0, which uses pedigree reconstruction to infer likelihood of genetic relationships, rather than a pairwise approach. Analyses were performed assuming female and male polygamy (per Marie et al., 2019), dioecious and diploid samples, a genotyping error rate of 0.01, and an absence of inbreeding or clones. All analyses were performed assuming the Full‐Likelihood (FL) method with high precision, sibship scaling, no updating of allele frequencies, and a weak sibship prior. Three runs were performed using the “very long” option in the program (with three different random number seeds) to ensure consistency of familial assignments; and for comparison purposes, three additional Colony runs were performed assuming duplicate parameters as outlined above, however, these runs assumed the presence of inbreeding. Relationships were only deemed “true” if parent‐offspring or sibling pairs were identified across all six Colony runs with >95% probability and were deemed “possible” if probabilities exceeded 90%. To support any inferred parent‐offspring and/or sibling relationships: (1) parentage exclusion probabilities were estimated using the program COANCESTRY 1.0.1.10 (Wang, 2011) and all adult Galápagos genotypes, and by assuming a genotyping error rate of 0.01 and (2) by estimating pairwise relatedness among all ETP sharks using COANCESTRY. To determine the most appropriate relatedness estimator for our dataset, we used the R package related (Pew et al., 2015) to simulate 1000 pairs of individuals for each of four relatedness groups (parent‐offspring, full‐siblings, half‐siblings, and unrelated) and four relatedness metrics (i.e., Li et al., 1993; Queller & Goodnight, 1989; Lynch & Ritland, 1999; Wang, 2002), and using ETP scalloped hammerhead microsatellite allele frequencies. Within the simulations, Wang (2002) possessed the highest correlation between the observed and expected relatedness values (*r* ^2^ = 0.82) and was therefore selected for use herein (data not shown). Overall mean relatedness (Wang, 2002) among individuals was calculated for combined ETP adults and YOYs. For each pair of colony‐identified putative parent‐offspring pairs, or full‐ or half‐sibling pairs, pairwise relatedness was estimated for comparison to the overall ETP mean value (see “Section 3”).

### 2.5 Mitochondrial control region sequencing and published data mining

The complete mitochondrial DNA control region (~1200‐bp) was amplified in 25 μl polymerase chain reactions (PCR) using the Forward and Reverse primers CR‐F6 and DAS‐R2 as well as the reaction conditions and amplification protocols outlined in Clarke et al. (2015). PCR purification and sequencing was performed by GENEWIZ, Inc. using Applied Biosystems BigDye version 3.1. Single‐strand sequencing of ~700‐bp of the 5′ end of the control region was performed using the Forward primer, CR‐F6, and an Applied Biosystem's 3730xl DNA Analyzer. The 5′ end of the control region was targeted for sequencing as a previous global scalloped hammerhead mitochondrial survey showed that most polymorphic sites are present within the first 548‐bp of this locus (Duncan et al., 2006).

All sequences were imported into Geneious 9.0.5 (Kearse et al., 2012) and chromatograms were visually inspected for base calling errors. Raw sequences were cropped to 548‐bp to correspond to the same region analyzed by several other scalloped hammerhead control region studies (Chapman et al., 2009; Duncan et al., 2006; Nance et al., 2011; Quintanilla et al., 2015). Prior to downstream population genetic analysis, species identity of all sequences was tested using the National Center for Biotechnology Information BLAST tool (Altschul et al., 1990), and any identified species misidentifications were discarded (along with its corresponding multi‐locus genotype if present within the microsatellite dataset).

To increase the mitochondrial sequence dataset for our ETP analyses, we added 63 published sequences (Quintanilla et al., 2015) of scalloped hammerheads sampled from the Colombian Pacific (this combined dataset hereafter referred to as ETP‐CR‐Expanded dataset). These 63 sequences (GenBank Accession numbers KM922592‐KM922595) represented adult sharks from Malpelo Island (MAL; *n* = 18), and YOY sharks from two nursery sites on the Colombian coast: Sanquianga (SAN; *n* = 22) and Tribugá (TRI; *n* = 23) (Figure 2; Table 1). While other published ETP scalloped hammerhead control region sequences exist (Duncan et al., 2006; Green et al., 2022; Nance et al., 2011), these were from unknown or mixed age class individuals, so we excluded them to maintain demographic consistency in our analyses (i.e., sharks that were unambiguously adults or YOY).

*Worldwide phylogeography samples:* We assessed scalloped hammerhead matrilineal evolutionary relationships worldwide by combining our ETP‐CR‐Expanded mitochondrial sequence dataset trimmed to match the same 515‐bp stretch of DNA sequence available from previously published control region‐based studies of this species worldwide, regardless of shark age class (published datasets from Duncan et al., 2006; Nance et al., 2011; Quintanilla et al., 2015; Spaet et al., 2015; Pinhal et al., 2020; Green et al., 2022—note: Mexico and Hawaii samples from Green et al. (2022) were not included given their sample overlap with Duncan et al.'s (2006) global *S. lewini* phylogeographic survey). Prior to analysis, the control region datasets from each of these published studies was first curated and reconstructed (i.e., frequency of recovered haplotypes determined), resulting in a worldwide dataset containing 1818 control region sequences (hereafter the Worldwide‐CR‐Dataset).

### 2.6 Mitochondrial DNA: Population structure and phylogeography

We compared levels of mitochondrial genetic diversity across scalloped hammerhead subpopulations (i.e., global adult samples [SEY, FLA, and GAL] and ETP subpopulations [ETP‐CR‐Expanded Dataset]) by estimating standard diversity indices (H, number of haplotypes; S, number of segregating sites; *h*, haplotype diversity; *π*, nucleotide diversity) using Arlequin 3.5 (Excoffier & Lischer, 2010). HACSim (Phillips et al., 2020) was used to plot haplotype accumulation curves. Pairwise estimates of matrilineal genetic differentiation were generated in Arlequin by means of Wright's pairwise fixation indices (*F*_ST_; Wright, 1965) and the distance‐based Φ_ST_ metric (Excoffier et al., 1992). Mantel tests were performed in Arlequin to test for isolation‐by‐distance (IBD) among ETP mainland coastal YOY nursery sites. Significance of the Mantel test was determined using 1000 permutations.

Evolutionary relationships among haplotypes at two different geographic scales were visualized via median‐joining haplotype networks (Bandelt et al., 1999) constructed using PopART (Leigh & Bryant, 2015): (1) Adult scalloped hammerhead global haplotype diversity and phylogeography were assessed via a network containing mitochondrial DNA sequences from the three adult subpopulations (SEY, FLA, and GAL). (2) Worldwide scalloped hammerhead historical matrilineal evolutionary relationships were assessed via a median‐joining network constructed from the Worldwide‐CR‐Dataset (*n* = 1818 sequences).

## 3 RESULTS

### 3.1 Microsatellite genotyping and mitochondrial control region sequencing—quality control and filtering

Across the entire genotyped dataset, six pairs of likely duplicate samples were found (i.e., individuals shared the same microsatellite profile save for two or less mismatched alleles); five of these six putative pairs were comprised of individuals sampled from the same geographic location and had the same mitochondrial control region haplotype, suggesting inadvertent duplicate sampling of YOY market‐derived sharks (no duplicates were found among adult samples). A single individual from each of these five pairs (i.e., the individual with the highest rate of missing data) was discarded from both control region and microsatellite datasets. The individuals comprising the sixth pair, however, were collected from different locations and processed at separate times in the laboratory so both were retained for analysis given the low likelihood of repeat sampling of the same individual and/or sample mix‐up. Species misidentifications in the field or sample labeling errors made during collection (see mitochondrial DNA results below) resulted in the discarding of another 30 individuals from the microsatellite dataset. Final sample sizes (*n* = 549) in Figure 2 and Tables 1 and S2 reflect the total number of microsatellite genotypes obtained after removal of sample duplicates and misidentifications.

Of the 10 microsatellite loci assessed, one locus (SLE018) demonstrated widespread evidence of null alleles, with 11 of the 14 sample collections exhibiting null allele frequencies greater than 10% (10.09–25.52%). Across the remaining nine loci, no single locus demonstrated a frequency of null alleles >10% in more than three subpopulations (Table S1). Hardy–Weinberg (HW) tests supported null allele findings, with locus SLE018 showing evidence of heterozygote deficits across 12 of 14 subpopulations at *p* < .05 after FDR correction. No other loci deviated significantly from HW expectations across subpopulations after FDR correction (Table S1). Linkage disequilibrium (LD) analysis identified four locus pairs that exhibited LD at *p* < .01 after FDR correction; however, there was no consistent pattern of disequilibrium between locus pairs across subpopulations, suggesting all loci segregated independently. Therefore, the remaining nine loci (SLE027, SLE033, SLE038, SLE045, SLE089, Cli‐12, SMO3, SMO7, and SMO8) were retained for statistical analysis. The final percentage of missing data for the nine‐locus microsatellite dataset was 5.55%. Loci were polymorphic in all locations and the number of alleles per locus ranged from 6–52 (Table S1). Both microsatellite datasets (global and ETP‐only) had sufficient statistical power with a 100% probability of detecting differentiation as low as *F*_ST_ = 0.003 (Figure S1). Mean overall allelic richness (*A*_R_) and the inbreeding coefficient (*F*_IS_) for all global samples collected for this study, including adults and YOY (*n* = 549), were 22.81 and 0.058, while observed (*H*_O_) and expected heterozygosity (*H*_E_) were 0.714, and 0.785, respectively.

Following duplicate and mis‐identified samples removal (see above), a final scalloped hammerhead control region sequence (548 bp) dataset from 515 individuals was obtained: 415 from the ETP (131 GAL adults plus 284 YOY), 50 adults from SEY, and 50 adults from FLA (Figure 2; Table 1). Of these 515 sequences, 22 segregating sites (15 transitions, seven transversions, and one indel) resolved a total of 20 haplotypes, eight novel to this study (GenBank Accession Numbers: OK082068‐OK082075). Rarefaction curves of haplotype accumulation for data generated herein showed that sampling effort captured 36.5% of the total haplotype variation in GAL, 95% in SEY, 56.2% in FLA, and 47.5% in the overall ETP (GAL adults and YOYs) (Figure S2A–D). Overall haplotype diversity (*h*) and nucleotide diversity (*π*) were 0.672 ± 0.016 and 0.0078 ± 0.0043, respectively.

### 3.2 Comparative genetic diversity and population structure of adult scalloped hammerheads from the Galápagos, Seychelles, and Florida

Nuclear genetic diversity was similar within the SEY and GAL collections (*A*_R_ = 12.0–12.7, *H*_O_ = 0.758–0.759, *H*_E_ = 0.759–0.796; Table 1), and lower within FLA adults (*A*_R_ = 9.5, *H*_O_ = 0.597, *H*_E_ = 0.612). Similarly, mitochondrial DNA yielded variable comparative estimates of diversity (Table 1), with haplotype diversity highest within adult sharks comprising the GAL aggregation (*h* = 0.550 ± 0.027) and the lowest within the SEY sharks (*h* = 0.184 ± 0.068). Nucleotide diversity was largely similar across sites (*π* = 0.0007–0.0012; Table 1).

**FIGURE 3:**
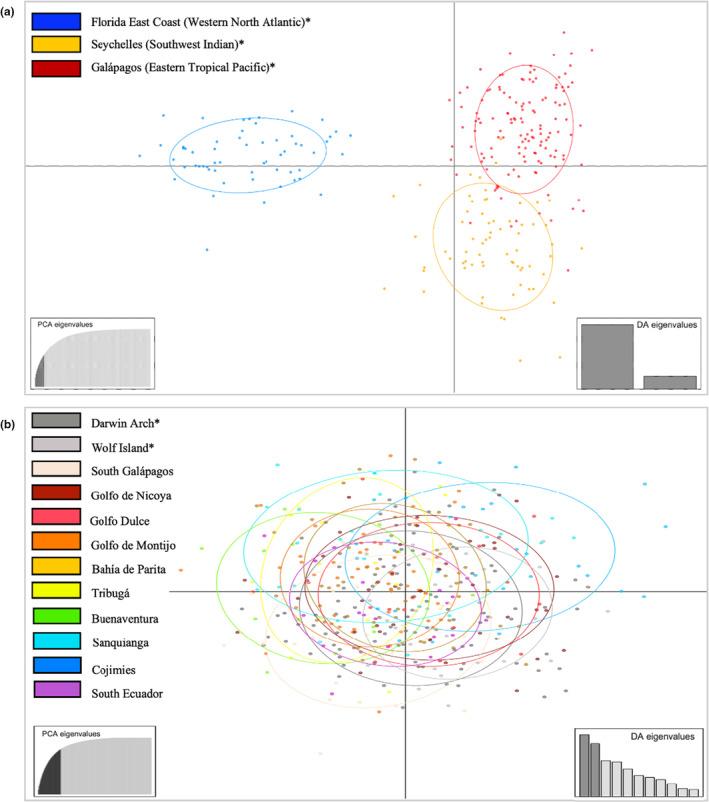
Discriminant analysis of principal components (DAPC) scatterplots of scalloped hammerhead sharks genotyped at nine microsatellite loci. *Indicates adult sharks. (a) Adult shark genotypes comprising samples from the Galápagos, Seychelles, and Florida subpopulations. Eleven PCs and two discriminant functions were retained to describe the relationships between clusters. Proportion of conserved variance was 0.484. (b) Shark genotypes collected from within the 12 Eastern Tropical Pacific subpopulations. Thirty‐four PCs and 11 discriminant functions were retained to describe the relationships between clusters. Proportion of conserved variance was 0.787.

Pairwise estimates of microsatellite differentiation (*F*_ST_, $G_{\mathrm{ST}}^{\prime \prime }$, *D*_EST_) identified large and statistically significant genetic differentiation between the three adult subpopulations (*F*_ST_ = 0.021–0.119, $G_{\mathrm{ST}}^{\prime \prime }$ = 0.162–0.670, *D*_EST_ = 0.131–0.583, *p* < .001). Genetic differentiation between sharks from the western Atlantic (FLA) and the two Indo‐Pacific locations was 2–3 times higher for *F*_ST_ and 4–5 times higher for $G_{\mathrm{ST}}^{\prime \prime }$ and *D*_EST_, than the differentiation between sharks from the Indian (SEY) and Pacific (GAL) Oceans (Table 2). Microsatellite multivariate cluster analysis supported the pairwise findings, with DAPC analysis (11 retained PCs per α‐score optimization) demonstrating separation of the genotypes into three distinct clusters; while the FLA cluster showed strong separation from the other sampling sites with no overlap, the GAL and SEY clusters showed less differentiation with some minor overlap of genotypes between the two groups (Figure 3a).

**TABLE 2:** *Sphyrna lewini* global (adults only) and Eastern Tropical Pacific (ETP) (adults and YOY), subpopulation‐level pairwise values of differentiation for nine microsatellite DNA markers (msat) (*F*_ST_, $G_{\mathrm{ST}}^{\prime \prime }$, *D*_EST_) and mitochondrial control region (CR) sequences (*F*_ST_, Φ_ST_)

Population comparison	msat *F*_ST_	msat $G_{\mathrm{ST}}^{\prime \prime }$	msat *D*_EST_	CR *F*_ST_	CR Φ_ST_
Global adults					
GAL vs. SEY	**0.021**	**0.162**	**0.131**	**0.584**	**0.953**
GAL vs. FLA	**0.119**	**0.670**	**0.583**	**0.529**	**0.951**
SEY vs. FLA	**0.102**	**0.614**	**0.531**	**0.738**	**0.781**
ETP only—All Groups					
DAR vs. WOL	0.006	0.003	0.002	−0.001	−0.003
DAR vs. SGA	0.004	−0.015	−0.011	**0.287**	**0.251**
DAR v. MAL	–	–	–	−0.028	−0.031
DAR vs. GNI	0.005	0.008	0.006	−0.014	−0.011
DAR vs. GDU	0.012	0.016	0.012	−0.031	−0.018
DAR vs. GMO	0.004	0.004	0.003	−0.007	−0.004
DAR vs. BPA	0.005	0.009	0.007	−0.012	−0.011
DAR vs. TRI	0.011	−0.001	−0.001	0.010	0.006
DAR vs. BVA	0.010	−0.020	−0.015	−0.039	−0.041
DAR vs. SAN	0.008	0.008	0.007	−0.009	−0.012
DAR vs. COJ	0.008	0.021	0.016	0.000	0.005
DAR vs. SEC	0.007	−0.008	−0.006	0.056	0.042
WOL v. SGA	0.009	0.006	0.005	**0.276**	**0.256**
WOL vs. MAL	–	–	–	−0.001	−0.019
WOL vs. GNI	0.010	0.025	0.019	−0.002	0.011
WOL vs. GDU	0.015	0.015	0.012	−0.018	0.018
WOL vs. GMO	0.007	0.005	0.004	−0.020	−0.017
WOL vs. BPA	0.007	0.000	−0.001	−0.014	−0.005
WOL vs. TRI	0.016	0.021	0.016	−0.026	−0.020
WOL vs. BVA	0.015	0.019	0.004	−0.045	−0.042
WOL vs. SAN	0.014	0.038	0.029	−0.020	−0.018
WOL vs. COJ	0.011	0.018	0.014	0.009	0.000
WOL vs. SEC	0.007	−0.031	−0.023	−0.010	−0.009
SGA vs. MAL	–	–	–	**0.507**	**0.454**
SGA vs. GNI	0.007	−0.001	0.000	**0.357**	**0.360**
SGA vs. GDU	0.016	0.030	0.023	**0.479**	**0.498**
SGA vs. GMO	0.007	0.001	0.000	**0.287**	**0.251**
SGA vs. BPA	0.007	−0.001	−0.001	**0.308**	**0.294**
SGA vs. TRI	0.013	−0.003	−0.003	**0.248**	**0.226**
SGA vs. BVA	0.014	−0.012	−0.009	**0.489**	**0.489**
SGA vs. SAN	0.011	0.014	0.010	**0.276**	**0.237**
SGA vs. COJ	0.013	0.031	0.024	**0.381**	**0.281**
SGA vs. SEC	0.008	−0.017	−0.013	0.227*	0.227*
MAL vs. GNI	–	–	–	−0.034	−0.033
MAL vs. GDU	–	–	–	−0.049	−0.042
MAL vs. GMO	–	–	–	−0.014	−0.015
MAL vs. BPA	–	–	–	−0.024	−0.028
MAL vs. TRI	–	–	–	0.016	0.000
MAL vs. BVA	–	–	–	−0.051	−0.058
MAL vs. SAN	–	–	–	−0.018	−0.031
MAL vs. COJ	–	–	–	−0.019	−0.013
MAL vs. SEC	–	–	–	0.090	0.060
GNI vs. GDU	0.018	0.051	0.039	−0.034	−0.027
GNI vs. GMO	0.008	0.019	0.015	−0.012	0.002
GNI vs. BPA	0.006	0.003	0.003	−0.017	−0.013
GNI vs. TRI	0.011	−0.013	−0.010	0.007	0.015
GNI vs. BVA	0.015	0.006	0.005	−0.046	−0.041
GNI vs. SAN	0.011	0.019	0.015	−0.012	−0.009
GNI vs. COJ	0.011	0.026	0.020	−0.004	0.011
GNI vs. SEC	0.013	0.028	0.021	0.061	0.072
GDU vs. GMO	0.014	0.027	0.021	−0.025	0.002
GDU vs. BPA	0.015	0.033	0.025	−0.035	−0.023
GDU vs. TRI	0.023	0.040	0.031	−0.007	0.029
GDU vs. BVA	0.018	−0.012	−0.009	−0.057	−0.041
GDU vs. SAN	0.021	0.059	0.045	−0.031	−0.014
GDU vs. COJ	0.014	0.004	0.003	−0.030	0.003
GDU vs. SEC	0.010	−0.044	−0.033	0.055	0.098
GMO vs. BPA	0.004	−0.007	−0.006	−0.018	−0.013
GMO vs. TRI	0.014	0.015	0.011	−0.014	−0.017
GMO vs. BVA	0.013	−0.004	−0.003	−0.049	−0.047
GMO vs. SAN	0.008	−0.001	−0.001	−0.016	−0.013
GMO vs. COJ	0.011	0.031	0.024	0.006	0.007
GMO vs. SEC	0.010	0.011	0.009	0.017	0.003
BPA vs. TRI	0.012	0.002	0.001	−0.007	−0.001
BPA vs. BVA	0.022	0.007	0.005	−0.048	−0.047
BPA vs. SAN	0.010	0.015	0.011	−0.017	−0.012
BPA vs. COJ	0.009	0.019	0.015	−0.002	0.006
BPA vs. SEC	0.010	0.013	0.010	0.033	0.035
TRI vs. BVA	0.014	0.019	0.014	−0.038	−0.039
TRI vs. SAN	0.010	0.034	0.026	−0.012	−0.008
TRI vs. COJ	0.018	0.029	0.022	0.015	−0.002
TRI vs. SEC	0.015	−0.008	−0.006	−0.017	−0.019
BVA vs. SAN	0.012	−0.028	−0.021	−0.044	−0.046
BVA vs. COJ	0.021	0.051	0.040	−0.024	−0.028
BVA vs. SEC	0.015	−0.015	−0.011	0.008	0.008
SAN vs. COJ	0.011	0.010	0.007	−0.003	0.000
SAN vs. SEC	0.012	0.002	0.002	0.021	0.016
COJ vs. SEC	0.015	0.030	0.023	0.075	0.033

*Note*: Bolded, underlined values indicate significance at *p* < .0001; Bolded only values indicate significance at *p* < .05 after false discovery rate correction.

Abbreviations: BPA, Bahía Parita; BVA, Buenaventura; COJ, Cojimies; DAR, Darwin Arch; FLA, Florida adults; GAL, Galápagos adults; GDU, Golfo Dulce; GMO, Golfo de Montijo; GNI, Golfo Nicoya; MAL, Malpelo Island; SAN, Sanquianga; SEC, South Ecuador; SEY, Seychelles adults; SGA, South Galápagos; TRI, Tribugá; WOL, Wolf Island.

* Indicates significance (*p* < .05) before false discovery rate correction, but not after.

**FIGURE 4:**
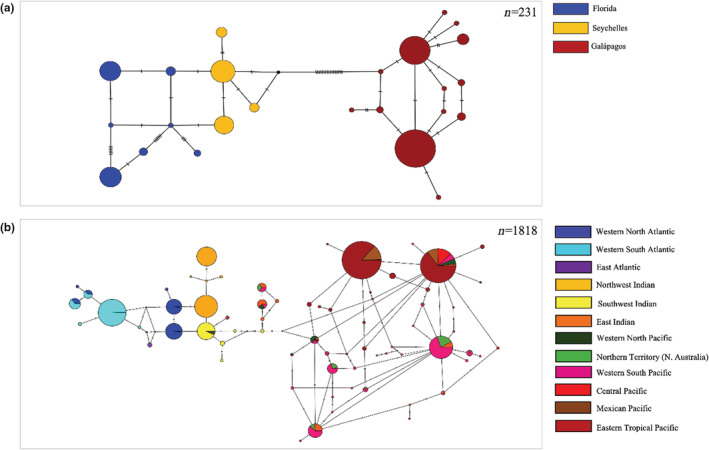
Global median‐joining haplotype networks of mitochondrial control region sequences of *Sphyrna lewini*. Size of circles is proportional to frequency of the haplotype. Black circles represent hypothetical missing haplotypes. (a) Median‐joining network of sequences from Florida (FLA), Seychelles (SEY), and Galápagos (GAL) adult sharks (*n* = 231; 548 bp). (b) Median‐joining network of sequences from our study and all available sequences from published *Sphyrna lewini* worldwide studies (*n* = 1818; 515 bp). Worldwide sample groupings of previously published data: Western North Atlantic (samples grouped from: East Coast USA, Gulf of Mexico, and Caribbean Sea; Duncan et al., 2006; Pinhal et al., 2020; this study); Western South Atlantic (samples grouped from Brazil: Para, Rio Grande do Norte, Rio de Janeiro, Sao Paulo, Santa Catarina, and Rio Grande do Sul; Duncan et al., 2006; Pinhal et al., 2020; this study); East Atlantic (West Africa; Duncan et al., 2006); Northwest Indian (samples grouped from: Arabian Sea and Red Sea; Spaet et al., 2015); Southwest Indian (samples grouped from: Seychelles and South Africa; Duncan et al., 2006; this study); East Indian (samples grouped from: West Australia, Indonesia, and Thailand; Duncan et al., 2006; Green et al., 2022); Western North Pacific (samples grouped from: Philippines and Taiwan; Duncan et al., 2006); Northern Territory (N. Australia) (Green et al., 2022); Western South Pacific (samples grouped from: Papua New Guinea, East Australia, Princess Charlotte Bay, Townsville, New South Wales, and Fiji; Duncan et al., 2006; Green et al., 2022); Central Pacific (Hawai'i; Duncan et al., 2006); Mexican Pacific (samples grouped from: Baja California, La Paz, and Mazatlan; Duncan et al., 2006; Nance et al., 2011); Eastern Tropical Pacific (samples grouped from: Costa Rica (Tarcoles, Golfo de Nicoya, Golfo Dulce), Panama (Pacific Panama, Golfo de Montijo, Chiriqui, Bahia de Parita), Colombia (Tribugá, Utria, Malpelo Island, Buenaventura, Sanquianga), Ecuador (Cojimies, South, Manta); Duncan et al., 2006; Nance et al., 2011; Quintanilla et al., 2015; this study); Galápagos (Darwin Arch, Wolf Island, South; this study).

Large and highly significant pairwise matrilineal differentiation was found among adult sharks from the GAL, SEY, and FLA (*F*_ST_ = 0.529–0.738, Φ_ST_ = 0.781–0.953, *p* < .00001; Table 2), along with strong phylogeographic structure and no haplotype sharing among sampling sites (Figure 4a). However, in contrast to the microsatellite patterns of geographic genetic differentiation (Figure 3a), the mitochondrial network indicated that recovered SEY and FLA haplotypes were more closely evolutionarily related than the SEY and GAL haplotypes (SEY and FLA clades were separated by only a single mutational step; the most closely related haplotypes between SEY and GAL were separated by 18 unsampled mutational steps).

### 3.3 Genetic diversity and population structure in the ETP

#### 3.3.1 Microsatellites

The overall microsatellite diversity estimates from nine loci (averaged) for 423 scalloped hammerheads comprising GAL adults and all YOY sampled from 12 ETP subpopulations were: *A*_R_ = 7.0, *H*_O_ = 0.762, and *H*_E_ = 0.75, with very few differences found among individual ETP sampling sites (*A*_R_ = 6.6–7.2, *H*_E_ = 0.730–0.768) (Table 1). Within the ETP, microsatellite pairwise estimates of *F*_ST_, $G_{\mathrm{ST}}^{\prime \prime }$, and *D*_EST_ identified no evidence of subpopulation differentiation between any of the surveyed locations (*F*_ST_ = 0.004–0.023, $G_{\mathrm{ST}}^{\prime \prime }$ = −0.044–0.059, *D*_EST_ = −0.021–0.045, *p* > .05; Table 2), and DAPC cluster analysis (34 PCs retained) also did not reveal any distinct separation and clustering of multi‐locus genotypes (Figure 3b). Microsatellite IBD analysis found no correlation between genetic and geographic distance among coastal mainland YOY nurseries (*r* = 0.272, *p* = .127).

#### 3.3.2 Mitochondrial DNA

Analysis of 478 control region sequences from 13 ETP locations (Adult and YOYs; Figure 2) yielded overall haplotype and nucleotide diversity estimates of 0.524 ± 0.016 and 0.001 ± 0.001, respectively. In addition, mitochondrial diversity estimates were largely similar among the ETP adult and YOY sampling sites (Table 1), save for within the YOY collection from the SGA, where only a single haplotype (the most common haplotype in this study) was recovered. There was no evidence of matrilineal genetic differentiation between the GAL aggregation adults and Malpelo Island (MAL) adults, nor between the ETP adult sharks and YOY sharks sampled from the nine mainland putative nursery areas. Furthermore, none of the YOY from the nine mainland nurseries were genetically differentiated from each other (Table 2). The only pairwise statistically significant differentiation found involved YOY sharks from the SGA nursery area—notably, these sharks (*n* = 31) were consistently differentiated from all other ETP subpopulations after FDR correction (save SEC, which showed differentiation before FDR; Table 2, S2). Mitochondrial DNA IBD analysis found no correlation between genetic and geographic distance among coastal mainland YOY nurseries (*r* = 0.000, *p* = .055).

An a posteriori analyses of pairwise genetic differentiation—incorporating available published data from all demographic groups—across the broader eastern Pacific (Mexico to Ecuador) and central Pacific (represented by Hawai'i sharks) showed no evidence of matrilineal population structure in the broader eastern Pacific, except for YOY from the South Galápagos (SGA) (as above), but significant differentiation between Hawaii and all broader eastern Pacific sharks (Table S2).

### 3.4 Relatedness of scalloped hammerheads within aggregations and nursery sites

No parent‐offspring‐pairs were reported by either program at any probability level; COANCESTRY estimated the probabilities of exclusion for the nine‐locus dataset as 1.06 × 10^−4^ and 2.72 × 10^−3^ when one parent or no parents of the offspring are known, respectively, and the probability of exclusion that a pair taken at random from the population are excluded as both parents was estimated as 1.48 × 10^−7^ for the dataset. Among the 134 genotyped adult female Galápagos aggregating sharks, no full‐sibling pairs were consistently identified across all six “very long" Colony runs with a probability exceeding 95%; however, one “possible” full‐sibling pair was identified in five of six runs at 91% probability (Table 3). Pairwise relatedness (Wang, 2002) of this “possible” adult pair was higher than the mean pairwise values found among ETP individuals (ETP mean = −0.0014; full‐sibling pair = 0.750; Table 3). This “possible” adult related pair consisted of one individual shark sampled at DAR and one individual sampled at WOL—sites separated by ~40 km. Each of these individuals was genotyped at eight of the nine microsatellite loci and both shared the same mitochondrial haplotype. No half‐sibling pairs were identified in the aggregation sharks.

**TABLE 3:** *Sphyrna lewini* Eastern Tropical Pacific full‐and half‐sibling pairs identified at probabilities greater than or equal to 90% across any of the six “very long” runs, along with the sibling pair's estimated pairwise relatedness per Wang (2002).

Relationship	Ind. 1	Capture location	Capture date	Ind. 2	Capture location	Capture date	Run 1 Prob.	Run 2 prob.	Run 3 Prob.	Run 4 prob.	Run 5 Prob.	Run 6 prob.	Wang (2002)
**Full‐Sibling**	**1262**	**COJ**	**07/04/2017**	**1270**	**COJ**	**07/04/2017**	**1.000**	**1.000**	**1.000**	**1.000**	**1.000**	**1.000**	**0.839**
**Full‐Sibling**	**1260**	**SEC**	**07/03/2017**	**1280**	**COJ**	**07/04/2017**	**0.997**	**0.997**	**0.997**	**0.997**	**0.997**	**0.997**	**0.715**
**Full‐Sibling**	**1265**	**COJ**	**07/04/2017**	**1269**	**COJ**	**07/04/2017**	**0.996**	**0.996**	**0.996**	**0.997**	**0.997**	**0.997**	**0.722**
Full‐Sibling	1401	TRI	06/27/2018	1404	TRI	06/27/2018	0.995	0.995	0.000	0.996	0.996	0.996	0.458
**Full‐Sibling**	**1252**	**SEC**	**07/03/2017**	**1257**	**SEC**	**07/03/2017**	**0.994**	**0.994**	**0.994**	**0.995**	**0.995**	**0.995**	**0.808**
Full‐Sibling	1271	COJ	07/04/2017	1273	COJ	07/04/2017	0.000	0.988	0.988	0.989	0.989	0.989	0.814
Full‐Sibling	1268	COJ	07/04/2017	1273	COJ	07/04/2017	0.000	0.961	0.961	0.961	0.961	0.961	0.393
Full‐Sibling	1263	COJ	07/04/2017	1267	COJ	07/04/2017	0.941	0.941	0.941	0.945	0.945	0.945	0.699
Full‐Sibling	1263	COJ	07/04/2017	1265	COJ	07/04/2017	0.924	0.924	0.924	0.930	0.930	0.930	0.462
Full‐Sibling	1040	DAR	08/21/2016	1108	WOL	12/13/2016	0.908	0.908	0.908	0.910	0.910	0.000	0.750
Half‐Sibling	1133	BPA	06/17/2017	1243	SEC	06/19/2017	0.000	0.000	0.000	0.946	0.946	0.946	0.045
Half‐Sibling	1296	SGA	01/21/2018	1320	SGA	05/25/2018	0.000	0.000	0.000	0.923	0.923	0.923	0.125
Half‐Sibling	1293	SGA	03/01/2013	1305	SGA	11/14/2018	0.912	0.912	0.912	0.915	0.915	0.915	0.334
Half‐Sibling	1297	SGA	03/28/2018	1306	SGA	03/21/2013	0.904	0.000	0.000	0.907	0.907	0.907	0.231
Half‐Sibling	1155	BPA	07/01/2017	1470	SAN	06/15/2013	0.903	0.903	0.903	0.000	0.000	0.000	0.272
Half‐Sibling	1256	SEC	07/03/2017	1312	SGA	06/18/2018	0.900	0.900	0.900	0.902	0.902	0.902	0.234

*Note*: Runs 1–3 were performed assuming no inbreeding, while runs 4–6 were performed assuming the presence of inbreeding. Bold values indicate pairs with probabilities >95% across all six runs.

Abbreviations: Ind., Individual; Date, Month/Day/Year; Prob., Probability. Locations: DAR, Darwin Arch; WOL, Wolf Island; SGA, South Galápagos; BPA, Bahía Parita; TRI, Utria‐Tribugá; SAN, Sanquianga; COJ, Cojimies; SEC, South Ecuador.

Among YOY ETP samples, Colony identified four full‐sibling pairs (comprising eight separate individuals) that were consistently detected across the six “very long" runs with probabilities greater than 95% (and two additional “possible” full‐siblings with probabilities >90%). Each of these pairs possessed pairwise estimates of relatedness that were higher than the overall mean value (Table 3), and three of the four full‐sibling pairs (>95%) were sampled from the same nursery sites within a single day of each other; all sibling pairs contained matching mitochondrial haplotypes. Notably, two of these pairs were sampled from the COJ nursery, one pair from the SEC nursery, and one pair contained individuals sampled from COJ and SEC. An additional two “possible” half‐sibling pairs—each with 90–91.2% probability across all six runs and higher than average pairwise relatedness estimates (Table 3)—were identified by Colony. Of these “possible” half‐sibling pairs, one pair contained two individuals collected from within the SGA nursery area in 2018. The other pair consisted of individuals sampled from geographically separate nurseries across different years—one individual sampled from SEC in 2017 and another sampled from the SGA in 2018. All four individuals that made up the two “possible” half‐sibling pairs shared the most common ETP mitochondrial haplotype.

### 3.5 Worldwide matrilineal phylogeography

A median‐joining network of scalloped hammerhead control region haplotype sequences (515 bp) from all age groups across 12 worldwide locations (i.e., Worldwide‐CR‐Dataset), illustrated three primary phylogeographic lineages consisting of samples from the: (1) Atlantic, (2) western Indian, and (3) eastern Indian‐Pacific regions with minor haplotype sharing between the latter two (Figure 4b). No phylogeographic partitioning was detected among haplotypes within the largely Pacific clade, with widespread sharing of the most common haplotypes occurring across the Pacific. Within the Atlantic, there was some separation between the western North and western South Atlantic, albeit with some haplotype sharing. The eastern Atlantic is nested within the greater Atlantic clade, however no haplotype sharing between the western and eastern Atlantic was observed.

## 4 DISCUSSION

This study complements and expands on previous studies of scalloped hammerheads from the ETP and broader eastern Pacific (Daly‐Engel et al., 2012; Duncan et al., 2006; Nance et al., 2011; Quintanilla et al., 2015) in three ways: (1) since different age classes of scalloped hammerheads have different migratory and thus gene flow capabilities, we chose to focus our analyses mainly on specific demographic groups to avoid potentially confounding influences in the biological inferences made. To this end, we focused our population genetic structure assessment of ETP sharks sampled across ~2000 km of continental coastline (Costa Rica to Ecuador) entirely on (a) highly site‐resident YOY animals captured in putative nursery areas, and separately on (b) confirmed adult sharks known to have long distance migratory capabilities; (2) we doubled the sample sizes used by the largest ETP study to date (Nance et al., 2011; *n* = 221), allowing increased confidence in study inferences; and (3) we provide the first population genetic characterization of a scalloped hammerhead aggregation, in this case the large and iconic aggregations that form at the northern Galápagos Darwin and Wolf Islands. We discuss our findings in the context of each study objective.

### 4.1 Comparative genetic diversity, population structure, and phylogeography of adult scalloped hammerheads

The GAL scalloped hammerhead aggregation adults had the highest mitochondrial control region haplotype diversity of the three adult populations surveyed herein, despite the haplotype accumulation curves indicating that our GAL sharks sampling captured less of the total genetic variation present here compared with sampling of SEY and FLA sharks (36.5% vs. 95% and 56.2%, respectively) (Figure S2A–D). This finding suggests that actual mitochondrial diversity may be higher in the Galápagos than reported in this study. Notably, the haplotype accumulation curve for SEY samples indicated that 95% of the variation present within the SEY was likely captured, despite the discovery of only two haplotypes. The GAL aggregation adults also demonstrated high or higher nuclear genetic diversity (microsatellite allelic richness [*A*_R_] and observed heterozygosity [*H*_O_]) compared with adults from the SEY and FLA, respectively. Furthermore, GAL adult samples also had much higher nuclear genetic diversity (*A*_R_ = 12.0; average number of alleles = 16.2; *H*_O_ = 0.759) than adult scalloped hammerheads sampled from the broader western Atlantic (*A*_R_ = 4.2–5.0; average number of alleles = 6.4; *H*_O_ = 0.52–0.64; Pinhal et al., 2020), and Malpelo Island in the ETP (average number of alleles = 6.7; *H*_O_ = 0.560; Quintanilla et al., 2015)—the only other studies reporting genetic diversity assessments for adult sharks. We note that inferences of genetic diversity in the Galápagos aggregation compared with adult sharks from these two other studies is caveated on the basis that the set of microsatellite loci used in these studies only partially overlap and contained variable sample sizes. Furthermore, since our samples from Florida and the Seychelles and the ones analyzed by Pinhal et al. (2020) and Quintanilla et al. (2015) were from non‐aggregating adults, our findings of the GAL adults having the highest genetic diversities may be influenced by the fact that we have compared aggregating adults with adults from different phases in the life‐cycle. However, the overall result provides grounds for some optimism about the comparative, current genetic status of the iconic Galápagos aggregation, despite the high levels of IUU fishing in the ETP. To our knowledge, this is the first focused assessment of comparative genetic diversity in globally sourced, exclusively adult scalloped hammerheads—a key demographic group that forms the reproductive stock of this Critically Endangered species.

The strong nuclear and mitochondrial pairwise genetic differentiation observed among adult scalloped hammerheads from the GAL, SEY, and FLA, along with the distinct, ocean basin, matrilineal phylogeographic lineages they form with no haplotype sharing (Figure 4a), is consistent with previous findings from analyses of albeit mixed‐age demographic groups from the Pacific, Indian and Atlantic Oceans (Daly‐Engel et al., 2012; Duncan et al., 2006; Quintanilla et al., 2015; Spaet et al., 2015). Our findings from the adult sharks reinforce the independent evolutionary trajectories of scalloped hammerheads in each of the three ocean basins, and consequently the importance of implementing conservation management efforts, at the very least on an ocean basin scale, to preserve the distinct lineages of this globally overfished species.

Our phylogeographic results showing Atlantic (FLA) and Indian Ocean (SEY) scalloped hammerheads as having the closest mitochondrial evolutionary relationship of the three adult populations assessed (Figure 4a) is consistent with the mixed‐age group, mitochondrial control region phylogeography reported by Duncan et al. (2006), Daly‐Engel et al. (2012) and Spaet et al. (2015), which show that the Indian/western Pacific/Atlantic sharks form the closest grouping relative to central/eastern Pacific sharks, likely due to historic, sporadic female dispersal events between the Atlantic and Indian Ocean around South Africa. Our finding of the Galápagos adults as the most divergent matrilineal lineage (separated by at least 18 mutational steps from Seychelles adults and at least 20 steps from western Atlantic adults) is concordant with Daly‐Engel et al.'s (2012) findings of the largest population differentiation of scalloped hammerhead globally occurring between the ETP and the western Atlantic populations.

In contrast to the mitochondrial DNA results, nuclear differentiation between the Seychelles and Galápagos adults was much smaller than between these two populations and Florida adults. This contrast in differentiation between the two organelle markers may be attributed to their mutation rate and mode of inheritance. Biparentally inherited microsatellites mutate faster than matrilineally inherited mtDNA, and can be used to detect population structure, or conversely male‐mediated gene flow, on a relatively contemporary timescale (10–100 generations ago) (Selkoe & Toonen, 2006). Microsatellite‐based, contemporary male‐mediated gene flow in scalloped hammerheads across vast oceanic distances has been suggested by the lack of observed structure between the western Indian and central Pacific Oceans, and even (albeit at a lower level) between the ETP and central Pacific (Daly‐Engel et al., 2012). Our results showing statistically significant but lower level of nuclear differentiation between Seychelles and Galápagos adults are consistent with the scenario proposed by Daly‐Engel et al. (2012), reflecting some contemporary dispersal across the Indo‐Pacific biogeographic provinces, including between the east to west Indo‐Pacific range of the scalloped hammerhead.

### 4.2 Genetic diversity, population structure and philopatry in the ETP

Nuclear and mitochondrial genetic diversity estimates (Nuclear: Avg. *H*_O_ = 0.762; mitochondrial: *h* = 0.524; *π* = 0.0011) in the ETP scalloped hammerheads (both YOY and adults collected for the present study), were similar to those reported by Nance et al. (2011) (Avg. *H*_O_ = 0.77; *h* = 0.53; *π* = 0.0011) for the same general geographic region, even though our sample size was nearly twice as large. The similar values from two studies using different sample sets suggest that these estimates are likely reliable indicators of genetic diversity of scalloped hammerheads in the ETP biogeographic region. Akin to our findings from Galápagos adults, the overall nuclear diversity estimated across all demographic groups sampled in the ETP (Avg. *H*_O_ = 0.76–0.77; values from our study and Nance et al., 2011) is on the high side of the values reported across all published studies of scalloped hammerheads from other parts of its global distribution (other studies *H*_O_ = 0.58–0.79; Ovenden et al., 2011; Daly‐Engel et al., 2012; Spaet et al., 2015; Pinhal et al., 2020; Green et al., 2022), lending further support to the notion that genetic diversity in the ETP still remains comparatively high. Notably, the average *H*_O_ values for ETP scalloped hammerheads in our study were also higher than average *H*_O_ values of 20 of the 28 shark species reviewed by Domingues et al. (2018).

Our findings of little genetic differentiation among the scalloped hammerhead adults and YOY in the ETP, coupled with geographically widespread mitochondrial control region haplotype sharing and no evidence of IBD among YOY sampled along the mainland putative nurseries, supports the presence of high genetic connectivity in this region, and is inconsistent with a hypothesis of philopatric behavior by females in this region. Rather, our data are consistent with studies suggesting females of this species stray between coastally connected nursery areas for parturition (Duncan et al., 2006; Quintanilla et al., 2015). Our findings related to population structure in the ETP, however, differ somewhat from those reported by Nance et al. (2011)—the only other study for this biogeographic region generally similar to ours in terms of spatial sampling scale and markers used. Nance et al. (2011) found subtle, but statistically significant, microsatellite genetic differentiation at population‐level (global *F*_ST_ = 0.005, *p* < .001), but not individual level analyses, across seven coastal sampling sites spanning Baja, Mexico to Ecuador. While they also found little pairwise genetic differentiation across their sampling sites using mitochondrial control region sequences, they did detect a marginally significant IBD signal. Given these marked inconsistent results and only subtle statistical differentiation where it existed, Nance et al. (2011) acknowledged uncertainty in the biological significance of their population structure results. We suggest that given the twice as large sample set (albeit our nine vs. their 15 microsatellites) that included a large number of adults from offshore islands in our study, and Nance et al.'s mixed population structure results, the most parsimonious inference is that scalloped hammerheads in the ETP comprise a single genetic stock. Notably, and akin to the observations of Duncan et al. (2006) and Daly‐Engel et al. (2012) (albeit made with much smaller sample sizes that were primarily from Mexico), we report high differentiation (Avg. *F*_ST_ = 0.523; Avg. Φ_ST_ = 0.542) between scalloped hammerheads from the ETP and Hawai'i (Table S2), making it highly likely that strong matrilineal differentiation in this species is the true state of nature across this Eastern‐central Pacific ocean expanse.

Although general trends in our data indicate high connectivity in the ETP, we note that significant mitochondrial, but no nuclear, differentiation was consistently detected between one putative nursery site—the South Galápagos (SGA)—and all other sampling locations save the South Ecuador (SEC) nursery. This marker disparity may reflect the differences in mitochondrial versus microsatellite loci evolutionary rates, but also keeps open the possibility that some scalloped hammerhead females show philopatry to this insular nursery site or the stochastic use of this site by only a small subset of females. Temporal sampling of sharks at this Galápagos nursery is needed to resolve if YOY here represent a differentiated population.

While our results do not support female philopatry to nursery sites along the length of ETP continental coastline examined here, regional philopatry in scalloped hammerheads has been inferred from matrilineal population structure in some other parts of the species distribution. Indeed, Rangel‐Morales et al. (2022), utilizing whole mitochondrial genome sequences and microsatellites to analyze YOY scalloped hammerheads sampled along much of the Mexican Pacific coast found mitochondrial but not nuclear differentiation, and suggested regional female philopatry to nursery areas to explain their findings. Philopatric behavior by scalloped hammerhead females has also been proposed over continuous coastline in the western Atlantic (Chapman et al., 2009; Pinhal et al., 2020), and across discontinuous coastlines globally (Daly‐Engel et al., 2012). Matrilineal population structure across the scalloped hammerhead's extensive latitudinal distribution in the western Atlantic (~41°N to 34°S; Rigby et al., 2019) could also result from either the massive geographic distances involved or from potential soft restrictions caused by oceanographic factors not present in the species' much more compact latitudinal range (~32°N to ~6°S) in the ETP. Indeed, the western Atlantic Ocean has been classified into three biogeographic realms based on their high species endemicity (Costello et al., 2017), whereas the ETP is considered a single biogeographic realm with few geographic barriers to gene flow (Costello et al., 2017; Floeter et al., 2008; Kulbicki et al., 2013).

### 4.3 Relatedness of scalloped hammerheads within aggregations and nursery sites

The northern Galápagos Islands boast the highest shark biomass per area in the world, with more than half of this biomass comprising the iconic scalloped hammerhead aggregations (Salinas‐de‐León et al., 2016). How the sharks annually locate these remote offshore sites during their migrations remains unclear. We investigated kinship within the scalloped hammerhead adults at the aggregations to determine whether familial relationships might play a role in where these sharks aggregate. There have been very few investigations of kinship within aggregating elasmobranchs, with patterns so far appearing to be species‐specific. A study on migratory basking sharks (*Cetorhinus maximus*) which aggregate seasonally in the Northeast Atlantic demonstrated that within group relatedness was higher than expected by chance, especially among the females (Lieber et al., 2020). In contrast, Venables et al. (2021) found no evidence that reef manta rays (*Mobula alfredi*) aggregating at sites in the western Indian Ocean were more related than expected by chance and suggested that kinship did not play a role in visits to aggregation sites. Furthermore, no correlation between kinship and social networks (although not technically aggregations) was found in blacktip reef sharks (*Carcharhinus melanopterus*) in French Polynesia (Mourier & Planes, 2021). In our study of the aggregating hammerhead adults, only a single “possible” full‐sibling and no half‐sibling pairs were identified, suggesting that relatedness is not a driver of how individuals choose the Wolf and Darwin Islands aggregation sites. Some shark species create cognitive maps using the earth's magnetic field to navigate back to locations with prey availability (Keller et al., 2021; Kimber et al., 2014; Meyer et al., 2010), and scalloped hammerheads are hypothesized to use geomagnetic topotaxis for navigation between seamounts (Klimley, 1993). It is possible that once a topographically and environmentally suitable insular aggregation site is discovered during adult migrations, the scalloped hammerheads remember and repeatedly return to the aggregation site for the social interaction and/or foraging benefits it provides.

Unlike Quintanilla et al. (2015) who found parent‐offspring relationships between adult sharks sampled at Malpelo Island and two coastal Colombian nurseries, our investigation of kinship between Galápagos aggregations and YOY from widely distributed coastal nursery sites (north Costa Rica to south Ecuador), however, yielded no parent‐offspring relationships. High connectivity and shared haplotypes between the Galápagos aggregation and coastal nurseries, as well as telemetry tracking data showing females from the Galápagos traveling to coastal Panama and back (P. Salinas de León, unpublished), however, indicate that parturition in these areas is possible. Although sample sizes analyzed in this study were the largest from the ETP to date, the number of sharks present in the aggregations and nursery sites is not known but is likely to be large based on the high frequency of YOY sharks found in artisanal markets (Guzman et al., 2020; O'Bryhim et al., 2021). It is possible, therefore, that the failure to find parent‐offspring pairs in our study is a result of insufficient sampling of YOY sharks. Likewise, we found only four high probability full‐siblings and no‐half sibling pairs across 289 genotyped YOYs. While kinship results in this study provide a preliminary view of the reproductive behavior of scalloped hammerheads in the ETP, further insight will require more exhaustive sampling of YOYs and the use of a larger marker set.

### 4.4 Worldwide matrilineal phylogeography

To add to existing matrilineal phylogeographic hypotheses about scalloped hammerheads worldwide (Duncan et al., 2006; Fields et al., 2020; Green et al., 2022; Quintanilla et al., 2015; Spaet et al., 2015), we added our 515 mtCR sequences (from the ETP, Seychelles and Florida) to published data from other global collection sites increasing the global dataset by almost a third, thus providing a phylogeographic view based on the largest dataset to date (1818 individual sequences). Scalloped hammerheads clustered primarily into three phylogeographic lineages corresponding to the Atlantic, western Indian, and eastern Indian‐Pacific regions with minor haplotype sharing between the latter two. The observed pattern of ocean basin lineage relationships derived from this much larger sequence dataset remain concordant with previous hypotheses of closer evolutionary relationships between scalloped hammerheads in the Atlantic and Indian Ocean, relative to the Pacific lineage. We note that the addition of 415 sequences from the ETP revealed haplotype sharing between scalloped hammerheads from the western and eastern Pacific, as one of the most common haplotypes is represented in all Pacific sampling locations. Although multiple low frequency haplotypes were found to be unique to the ETP, more exhaustive sampling of the western Pacific is needed to determine whether these haplotypes are present but unsampled in other Pacific locations.

## 5 CONCLUSION AND MANAGEMENT IMPLICATIONS

This study represents the first genetic investigation of the iconic Galápagos scalloped hammerhead aggregation, and the largest sample size‐based investigation of population structure and phylogeography of this species in the ETP to date. Our results, using both matrilineal and nuclear genetic markers, indicate high connectivity between the Galápagos aggregation and all coastal nursery sites, and no convincing evidence of philopatry within the ETP. This extensive connectivity in a region known for high IUU fishing points to the need for coordinated, multinational management cooperation among ETP jurisdictions in order to conserve *Sphyrna lewini* in both the Galápagos aggregations and ETP as a whole. Additionally, we underscore the need for management strategies for the three evolutionarily unique (phylogeographic) lineages in each ocean basin order to conserve their biodiversity.

## AUTHOR CONTRIBUTIONS

**Sydney P. Harned:** Data curation (lead); formal analysis (lead); writing – original draft (lead). **Andrea M. Bernard:** Data curation (supporting); formal analysis (supporting); writing – original draft (supporting); writing – review and editing (lead). **Pelayo Salinas de Leon:** Conceptualization (lead); data curation (lead); writing – review and editing (supporting). **Marissa R. Mehlrose:** Data curation (supporting). **Jenifer Suarez:** Data curation (supporting); writing – review and editing (supporting). **Yolani Robles:** Data curation (supporting); writing – review and editing (supporting). **Sandra Bessudo:** Data curation (supporting); writing – review and editing (supporting). **Felipe Ladino:** Data curation (supporting); writing – review and editing (supporting). **Andrés López Garo:** Data curation (supporting); writing – review and editing (supporting). **Ilena Zanella:** Data curation (supporting); writing – review and editing (supporting). **Kevin A. Feldheim:** Formal analysis (supporting); writing – review and editing (supporting). **Mahmood S. Shivji:** Conceptualization (lead); data curation (supporting); writing ‐ original draft (supporting); writing ‐ review and editing (lead).

## CONFLICT OF INTEREST

The authors report no conflict of interest.

## Supporting information


Appendix S1:

## Data Availability

All mitochondrial data underlying our analyses can be accessed at https://www.ncbi.nlm.nih.gov/ (GenBank Accession Numbers: OK082068‐OK082075). Microsatellite genotypes are available from Dryad at https://doi.org/10.5061/dryad.7m0cfxpzr.
