# Supplementary material for: Genetic population dynamics of the critically endangered scalloped hammerhead shark (Sphyrna lewini) in the Eastern Tropical Pacific
Source: Ecol Evol. 2022 Dec 28;12(12):e9642. doi: 10.1002/ece3.9642 (PMC9797937; doi:10.1002/ece3.9642)
Supplement: Supplementary file 1 — Appendix S1: [file ECE3-12-e9642-s001.docx]

Online Supplementary Materials
 **Genetic population dynamics of the critically endangered scalloped hammerhead shark (*Sphyrna lewini*) in the Eastern Tropical Pacific**

^1^Sydney Harned (spharned@ncsu.edu)

^1^Andrea M. Bernard (andrbern@nova.edu)

^2^Pelayo Salinas de León (Pelayo.salinas@fcdarwin.org.ec)

^3^Jenifer Suarez (jmsuarez@galapagos.gob.ec)

^4^Yolani Robles (yolrobles@hotmail.com)

^5^Sandra Bessudo (sbessudo@fundacionmalpelo.org)

^5^Felipe Ladino (fladino@fundacionmalpelo.org)

^6^Andrés López Garo (alopezgarro@gmail.com)

^6^Ilena Zanella ([ilena.zanella@gmail.com](mailto:ilena.zanella@gmail.com))

^7^Kevin A. Feldheim ([kfeldheim@fieldmuseum.org](mailto:kfeldheim@fieldmuseum.org))

^1^Mahmood S. Shivji (mahmood@nova.edu)

^1^Save Our Seas Foundation Shark Research Center and Guy Harvey Research Institute, Nova Southeastern University, 8000 North Ocean Drive, Dania Beach, Florida, USA 33004.

^2^Charles Darwin Research Station, Charles Darwin Foundation, Puerto Ayora, Galápagos Islands, Ecuador.
 ^3^Direccion Parque Nacional Galápagos, Departamento de Ecosistemas Marinos, Isla Santa Cruz, Islas Galápagos, Ecuador.
 ^4^Universidad de Panamá, Centro Regional Universitario de Veraguas.
 ^5^Fundacion Malpelo y otros Ecosistemas Marinos, Bogotá, Colombia.
 ^6^Asociación Conservacionista Misión Tiburon, Playas del Coco, Carrillo, Guanacaste, Costa Rica.
 ^7^Pritzker Laboratory for Molecular Systematics and Evolution, Field Museum of Natural History, Chicago, Illinois, USA.

Table S1. Summary statistics for 10 microsatellite loci genotyped across sampling locations for scalloped hammerheads (*Sphyrna lewini*): number of individuals genotyped (*n*), number of alleles (*a*)*,* allelic richness (*A_R_*), allele size range (*as*), frequency of null alleles (*Null*), observed heterozygosity (*H*_O_), expected heterozygosity (*H*_E_), and probability of conformation to Hardy-Weinberg expectations (*HWE*).

| **Location** | **SLE018** | **SLE027** | **SLE033** | **SLE038** | **SLE045** | **SLE089** | **Cli-12** | **SMO3** | **SMO7** | **SMO8** |
| --- | --- | --- | --- | --- | --- | --- | --- | --- | --- | --- |
| **DAR** |  |  |  |  |  |  |  |  |  |  |
| *n* | 100 | 100 | 101 | 98 | 100 | 99 | 100 | 99 | 97 | 81 |
| *a* | 6 | 11 | 9 | 12 | 5 | 12 | 34 | 18 | 3 | 35 |
| *A_R_* | 4.677 | 5.223 | 5.873 | 6.062 | 3.871 | 7.231 | 12.691 | 8.655 | 2.103 | 11.438 |
| *as* | 224-274 | 438-476 | 252-290 | 428-474 | 420-428 | 194-226 | 218-302 | 232-302 | 244-256 | 210-352 |
| *Null* | **0.2216** | 0.0156 | 0.0024 | 0.0163 | 0 | 0 | 0 | 0 | 0. 0291 | 0 |
| *H*_O_ | 0.320 | 0.650 | 0.693 | 0.776 | 0.650 | 0.838 | 0.950 | 0.909 | 0.433 | 0.914 |
| *H*_E_ | 0.693 | 0.647 | 0.731 | 0.789 | 0.649 | 0.825 | 0.935 | 0.872 | 0.476 | 0.908 |
| *HWE* | *0.0000* | 0.5391 | 0.4534 | 0.4056 | 0.7301 | 0.4150 | 0.3728 | 0.7853 | 0.6244 | 0.7654 |
| **WOL** |  |  |  |  |  |  |  |  |  |  |
| *n* | 32 | 27 | 31 | 32 | 31 | 27 | 31 | 32 | 32 | 31 |
| *a* | 5 | 6 | 8 | 7 | 4 | 11 | 24 | 15 | 3 | 14 |
| *A_R_* | 4.246 | 4.480 | 5.885 | 5.458 | 3.791 | 7.896 | 13.678 | 8.841 | 2.312 | 9.992 |
| *as* | 224-242 | 438-476 | 254-290 | 436-474 | 420-428 | 194-226 | 218-290 | 260-306 | 244-256 | 210-336 |
| *Null* | **0.2098** | 0 | 0 | 0 | 0.0358 | 0 | 0 | 0 | 0.0932 | 0 |
| *H*_O_ | 0.344 | 0.741 | 0.677 | 0.781 | 0.613 | 0.926 | 1.000 | 0.969 | 0.313 | 0.905 |
| *H*_E_ | 0.694 | 0.611 | 0.746 | 0.741 | 0.671 | 0.836 | 0.940 | 0.842 | 0.439 | 0.881 |
| *HWE* | *0.0000* | 0.8721 | 0.5959 | 0.8045 | 0.7028 | 0.5410 | 0.6131 | 0.7682 | 0.1346 | 0.2972 |
| **SGA** |  |  |  |  |  |  |  |  |  |  |
| *N* | 30 | 31 | 31 | 31 | 31 | 30 | 31 | 31 | 30 | 30 |
| *a* | 4 | 7 | 8 | 7 | 4 | 8 | 25 | 12 | 2 | 24 |
| *A_R_* | 3.868 | 4.799 | 5.892 | 5.105 | 3.510 | 6.843 | 14.159 | 8.756 | 2.000 | 13.320 |
| *as* | 224-240 | 438-476 | 254-290 | 436-474 | 420-428 | 202-226 | 218-292 | 268-298 | 244-248 | 210-364 |
| *Null* | 0.0339 | 0.0061 | 0.0254 | 0.0541 | 0 | 0.0866 | 0 | 0 | 0.0459 | 0.0059 |
| *H*_O_ | 0.600 | 0.613 | 0.774 | 0.677 | 0.613 | 0.667 | 0.968 | 0.903 | 0.400 | 0.933 |
| *H*_E_ | 0.661 | 0.617 | 0.768 | 0.722 | 0.605 | 0.823 | 0.946 | 0.869 | 0.464 | 0.928 |
| *HWE* | 0.2180 | 0.3660 | 0.2892 | *0.0304* | 0.8531 | *0.0261* | 0.9123 | 0.3068 | 0.4490 | 0.3797 |
| **GNI** |  |  |  |  |  |  |  |  |  |  |
| *N* | 35 | 39 | 40 | 38 | 42 | 40 | 40 | 42 | 41 | 41 |
| *a* | 7 | 8 | 9 | 7 | 4 | 12 | 26 | 12 | 3 | 24 |
| *A_R_* | 5.132 | 5.552 | 6.059 | 5.287 | 3.320 | 7.928 | 12.981 | 7.106 | 2.244 | 11.280 |
| *as* | 224-274 | 438-476 | 252-290 | 436-472 | 420-428 | 182-226 | 218-286 | 268-302 | 244-256 | 210-362 |
| *Null* | **0.1117** | 0 | 0 | 0.0125 | 0 | 0.0090 | 0.0100 | 0 | 0.0217 | 0 |
| *H*_O_ | 0.514 | 0.795 | 0.850 | 0.737 | 0.738 | 0.800 | 0.925 | 0.833 | 0.463 | 0.927 |
| *H*_E_ | 0.716 | 0.707 | 0.785 | 0.773 | 0.602 | 0.845 | 0.933 | 0.816 | 0.499 | 0.899 |
| *HWE* | *0.0108* | 0.4138 | 0.5690 | 0.7412 | 0.3259 | 0.6006 | 0.2018 | 0.1244 | 0.8576 | 0.2549 |
| **GDU** |  |  |  |  |  |  |  |  |  |  |
| *N* | 16 | 16 | 16 | 15 | 16 | 16 | 16 | 16 | 15 | 16 |
| *a* | 5 | 5 | 6 | 6 | 5 | 10 | 16 | 13 | 3 | 16 |
| *A_R_* | 4.852 | 4.474 | 5.202 | 5.274 | 4.567 | 7.732 | 12.285 | 9.930 | 2.667 | 11.979 |
| *as* | 224-240 | 438-476 | 268-290 | 436-474 | 420-428 | 192-226 | 218-278 | 260-298 | 244-256 | 210-340 |
| *Null* | **0.2552** | 0 | 0 | 0 | **0.1736** | 0.0122 | 0 | 0.0121 | 0.0670 | 0.0003 |
| *H*_O_ | 0.313 | 0.625 | 0.750 | 0.667 | 0.438 | 0.750 | 0.938 | 0.875 | 0.400 | 0.875 |
| *H*_E_ | 0.732 | 0.564 | 0.721 | 0.700 | 0.691 | 0.813 | 0.904 | 0.840 | 0.518 | 0.900 |
| *HWE* | *0.0000* | 0.8895 | 0.5393 | 0.8127 | 0.0023 | 0.7895 | 0.5726 | 0.9360 | 0.3986 | 0.6778 |
| **GMO** |  |  |  |  |  |  |  |  |  |  |
| *N* | 49 | 50 | 49 | 45 | 50 | 45 | 47 | 49 | 49 | 43 |
| *a* | 6 | 10 | 8 | 9 | 5 | 13 | 23 | 13 | 2 | 29 |
| *A_R_* | 4.505 | 5.403 | 6.112 | 6.357 | 4.313 | 8.143 | 11.343 | 8.159 | 2.000 | 12.828 |
| *as* | 224-274 | 438-476 | 270-290 | 438-480 | 420-428 | 194-228 | 218-296 | 264-298 | 244-248 | 210-364 |
| *Null* | **0.1782** | 0 | 0.0041 | 0.0209 | 0.0105 | 0 | 0 | 0 | 0 | 0 |
| *H*_O_ | 0.408 | 0.740 | 0.735 | 0.756 | 0.640 | 0.867 | 0.957 | 0.898 | 0.490 | 0.977 |
| *H*_E_ | 0.696 | 0.694 | 0.765 | 0.802 | 0.688 | 0.842 | 0.914 | 0.857 | 0.475 | 0.923 |
| *HWE* | *0.0000* | 0.5862 | 0.2970 | 0.5859 | 0.5981 | 0.6932 | 0.5437 | 0.3495 | 1.0000 | 1.0000 |
| **BPA** |  |  |  |  |  |  |  |  |  |  |
| *N* | 45 | 50 | 50 | 48 | 50 | 46 | 48 | 49 | 47 | 43 |
| *a* | 6 | 10 | 10 | 7 | 5 | 12 | 24 | 14 | 2 | 25 |
| *A_R_* | 4.558 | 5.732 | 6.871 | 5.521 | 4.129 | 7.723 | 12.991 | 8.114 | 2.000 | 11.691 |
| *as* | 224-274 | 438-478 | 254-290 | 436-472 | 420-428 | 194-226 | 218-296 | 260-302 | 244-248 | 210-364 |
| *Null* | **0.1576** | 0 | 0 | 0.0066 | 0 | 0.0223 | 0 | 0.0005 | 0 | 0 |
| *H*_O_ | 0.467 | 0.720 | 0.800 | 0.771 | 0.660 | 0.848 | 0.958 | 0.857 | 0.596 | 0.953 |
| *H*_E_ | 0.726 | 0.675 | 0.789 | 0.767 | 0.676 | 0.841 | 0.940 | 0.858 | 0.462 | 0.904 |
| *HWE* | *0.0000* | 0.9997 | 0.9798 | 0.6628 | 0.0628 | 0.8248 | 0.8698 | 0.3379 | 0.0643 | 0.5404 |
| **TRI** |  |  |  |  |  |  |  |  |  |  |
| *N* | 13 | 13 | 10 | 13 | 13 | 13 | 12 | 13 | 13 | 13 |
| *a* | 5 | 5 | 5 | 6 | 4 | 9 | 14 | 8 | 3 | 11 |
| *A_R_* | 4.761 | 4.537 | 5.000 | 5.676 | 3.769 | 8.022 | 12.578 | 7.445 | 2.954 | 9.523 |
| *as* | 224-240 | 438-476 | 270-290 | 436-474 | 422-428 | 198-226 | 228-286 | 270-298 | 244-256 | 210-276 |
| *Null* | **0.1927** | 0.0472 | 0 | 0 | 0 | 0 | 0.0068 | 0 | 0.0005 | 0.0193 |
| *H*_O_ | 0.385 | 0.615 | 0.700 | 0.923 | 0.692 | 0.923 | 0.833 | 1.000 | 0.462 | 0.769 |
| *H*_E_ | 0.716 | 0.675 | 0.690 | 0.749 | 0.592 | 0.820 | 0.899 | 0.834 | 0.462 | 0.852 |
| *HWE* | 0.0276 | 0.7373 | 0.4658 | 0.7257 | 0.7051 | 0.2585 | 0.0611 | 0.6558 | 1.000 | 0.1481 |
| **BVA** |  |  |  |  |  |  |  |  |  |  |
| *N* | 11 | 13 | 13 | 13 | 13 | 12 | 10 | 12 | 12 | 12 |
| *a* | 4 | 5 | 6 | 7 | 5 | 7 | 11 | 9 | 2 | 11 |
| *A_R_* | 3.909 | 4.715 | 5.531 | 6.261 | 4.754 | 6.829 | 11.000 | 8.453 | 2.000 | 9.933 |
| *as* | 224-240 | 438-476 | 270-290 | 436-474 | 420-428 | 204-226 | 232-274 | 270-306 | 244-248 | 210-340 |
| *Null* | **0.1817** | 0 | 0 | 0.0837 | 0 | 0 | 0.003 | 0.0003 | 0 | 0 |
| *H*_O_ | 0.364 | 0.615 | 0.769 | 0.615 | 0.615 | 0.833 | 0.900 | 0.833 | 0.667 | 0.917 |
| *H*_E_ | 0.682 | 0.636 | 0.731 | 0.737 | 0.686 | 0.840 | 0.875 | 0.844 | 0.486 | 0.813 |
| *HWE* | 0.0623 | 0.2036 | 0.9452 | 0.0674 | 0.2036 | 0.7366 | 0.7705 | 0.5793 | 0.5469 | 0.9395 |
| **SAN** |  |  |  |  |  |  |  |  |  |  |
| *N* | 16 | 24 | 24 | 20 | 21 | 19 | 24 | 24 | 23 | 24 |
| *a* | 6 | 8 | 6 | 8 | 5 | 11 | 22 | 13 | 2 | 17 |
| *A_R_* | 5.532 | 5.784 | 4.886 | 7.064 | 4.177 | 8.547 | 13.469 | 8.905 | 2.000 | 10.071 |
| *as* | 224-274 | 438-476 | 270-290 | 436-474 | 420-428 | 194-226 | 218-288 | 262-306 | 244-248 | 210-344 |
| *Null* | **0.1578** | 0.0700 | 0 | 0.0505 | 0 | 0 | 0 | 0 | 0 | 0 |
| *H*_O_ | 0.500 | 0.542 | 0.792 | 0.750 | 0.762 | 0.947 | 0.958 | 0.875 | 0.609 | 1.000 |
| *H*_E_ | 0.762 | 0.675 | 0.686 | 0.830 | 0.645 | 0.853 | 0.931 | 0.845 | 0.491 | 0.850 |
| *HWE* | **0.0102** | **0.0038** | 0.9255 | **0.0054** | 0.2438 | 0.5327 | 0.7519 | 0.5870 | 0.4039 | 0.8117 |
| **COJ** |  |  |  |  |  |  |  |  |  |  |
| *N* | 27 | 27 | 28 | 28 | 27 | 28 | 28 | 27 | 27 | 23 |
| *a* | 5 | 6 | 9 | 7 | 4 | 10 | 25 | 9 | 4 | 15 |
| *A_R_* | 4.350 | 4.914 | 6.533 | 5.335 | 3.865 | 7.254 | 14.787 | 6.569 | 2.948 | 10.212 |
| *as* | 224-274 | 438-476 | 260-290 | 436-496 | 420-428 | 190-214 | 218-290 | 270-294 | 242-256 | 210-350 |
| *Null* | **0.2245** | 0 | 0 | 0.0434 | 0 | 0 | 0 | 0.0944 | 0 | 0 |
| *H*_O_ | 0.357 | 0.704 | 0.750 | 0.607 | 0.741 | 0.821 | 0.893 | 0.630 | 0.667 | 0.913 |
| *H*_E_ | 0.746 | 0.629 | 0.744 | 0.756 | 0.641 | 0.811 | 0.948 | 0.764 | 0.538 | 0.888 |
| *HWE* | *0.0000* | 0.2457 | 0.5921 | 0.8861 | 0.8565 | *0.0034* | *0.0007* | 0.3401 | 0.4134 | 0.4323 |
| **SEC** |  |  |  |  |  |  |  |  |  |  |
| *N* | 22 | 21 | 22 | 19 | 20 | 21 | 21 | 22 | 22 | 21 |
| *a* | 6 | 8 | 6 | 9 | 4 | 10 | 19 | 13 | 3 | 19 |
| *A_R_* | 5.320 | 5.841 | 4.449 | 6.534 | 3.746 | 7.996 | 12.747 | 9.564 | 2.476 | 10.071 |
| *as* | 224-274 | 438-476 | 270-290 | 436-474 | 422-428 | 190-226 | 226-292 | 270-298 | 242-256 | 210-352 |
| *Null* | **0.1913** | 0 | 0.0165 | 0.0155 | 0.0005 | 0 | 0.0002 | 0 | 0 | 0 |
| *H*_O_ | 0.429 | 0.667 | 0.682 | 0.789 | 0.550 | 0.905 | 1.000 | 0.773 | 0.364 | 0.892 |
| *H*_E_ | 0.755 | 0.633 | 0.664 | 0.765 | 0.654 | 0.830 | 0.915 | 0.848 | 0.447 | 0.914 |
| *HWE* | *0.0007* | 0.7021 | 0.9289 | 0.4333 | 0.5187 | 0.1442 | 0.9963 | 0.5086 | 0.6794 | 0.9603 |
| **FLA** |  |  |  |  |  |  |  |  |  |  |
| *N* | 54 | 57 | 48 | 56 | 44 | 57 | 50 | 56 | 56 | 52 |
| *a* | 5 | 8 | 3 | 6 | 3 | 8 | 36 | 14 | 3 | 14 |
| *A_R_* | 4.815 | 7.610 | 3.000 | 5.785 | 3.000 | 7.895 | 34.433 | 12.916 | 2.956 | 13.666 |
| *as* | 202-242 | 446-490 | 270-274 | 439-466 | 422-428 | 196-216 | 226-302 | 270-310 | 248-258 | 210-270 |
| *Null* | 0 | 0.0241 | 0 | 0 | **0.1659** | 0 | 0 | 0 | 0 | **0.1140** |
| *H*_O_ | 0.278 | 0.614 | 0.250 | 0.786 | 0.227 | 0.772 | 0.960 | 0.946 | 0.161 | 0.654 |
| *H*_E_ | 0.283 | 0.606 | 0.227 | 0.710 | 0.432 | 0.683 | 0.960 | 0.860 | 0.150 | 0.879 |
| *HWE* | 0.2975 | 0.5487 | 1.0000 | 0.7986 | *0.0002* | 0.1661 | 0.6911 | 0.0767 | 1.0000 | 0.0000 |
| **SEY** |  |  |  |  |  |  |  |  |  |  |
| *N* | 64 | 64 | 57 | 68 | 60 | 65 | 64 | 63 | 64 | 53 |
| *a* | 7 | 10 | 11 | 9 | 5 | 11 | 21 | 21 | 6 | 37 |
| *A_R_* | 6.994 | 9.353 | 10.263 | 8.171 | 4.991 | 10.539 | 19.531 | 18.942 | 5.279 | 34.783 |
| *as* | 224-274 | 426-478 | 252-290 | 410-496 | 422-428 | 188-226 | 218-280 | 260-306 | 244-258 | 208-368 |
| *Null* | **0.1009** | 0.0132 | 0.0104 | 0 | 0 | 0 | 0.0859 | 0 | 0.0242 | 0.0319 |
| *H*_O_ | 0.531 | 0.766 | 0.667 | 0.824 | 0.667 | 0.877 | 0.750 | 0.905 | 0.469 | 0.868 |
| *H*_E_ | 0.723 | 0.793 | 0.732 | 0.823 | 0.654 | 0.867 | 0.914 | 0.909 | 0.517 | 0.956 |
| *HWE* | *0.0052* | *0.0133* | 0.4099 | 0.5251 | 0.1569 | 0.1910 | *0.0064* | 0.1218 | 0.6577 | 0.0602 |
| **All** |  |  |  |  |  |  |  |  |  |  |
| *n* | 514 | 532 | 520 | 524 | 519 | 519 | 522 | 535 | 528 | 473 |
| *a* | 10 | 19 | 14 | 18 | 6 | 20 | 43 | 28 | 8 | 52 |
| *A_R_* | 10 | 18.518 | 13.901 | 17.397 | 6.000 | 19.629 | 42.691 | 27.380 | 7.864 | 52.000 |
| *As* | 202-274 | 426-490 | 252-290 | 410-496 | 420-428 | 182-228 | 218-306 | 232-310 | 242-258 | 208-368 |
| *Null* | **0.1750** | 0.0376 | 0.0504 | 0.0415 | 0.0135 | 0.0179 | 0.0133 | 0.0024 | 0.0406 | 0.0194 |
| *H*_O_ | 0.409 | 0.686 | 0.690 | 0.760 | 0.618 | 0.836 | 0.925 | 0.884 | 0.445 | 0.890 |
| *H*_E_ | 0.705 | 0.738 | 0.782 | 0.833 | 0.658 | 0.865 | 0.949 | 0.873 | 0.518 | 0.931 |
| *HWE* | *0.0000* | *0.0000* | *0.0000* | *0.0000* | 0.5991 | 0.1381 | *0.0000* | 0.2199 | 0.2312 | 0.1414 |

**Bold** values represent null allele frequencies of >10% — FreeNA. Underlined values indicate null allele frequencies of >10% — MicroChecker. *Italicized* values indicate p-values significant after FDR correction.

Subpopulation Abbreviations: DAR, Darwin Arch; WOL, Wolf Island; SGA, South Galápagos; GNI, Golfo Nicoya; GDU, Golfo Dulce; GMO, Golfo de Montijo; BPA, Bahía Parita; TRI, Tribugá; BVA, Buenaventura; SAN, Sanquianga; COJ, Cojimies; SEC, South Ecuador; FLA, Florida East Coast; SEY, Seychelles; All, all populations.

Table S2. Pairwise *F*_ST_ (below) and *Φ*_ST_ (above) for 548-bp mitochondrial control region sequences available from all published *Sphyrna lewini* (all demographic groups) sampled in the Eastern Tropical Pacific and Central Pacific (Hawaii). Bold values indicate significance (p<0.05) after false discovery rate correction.

| Subpopulation | HAW | MEX | DAR | WOL | SGA | NCR | SCR | PAN | MAL | TRI | BVA | SAN | ECU |
| --- | --- | --- | --- | --- | --- | --- | --- | --- | --- | --- | --- | --- | --- |
| HAW (n=44) | - | **0.499** | **0.387** | **0.594** | **0.947** | **0.386** | **0.464** | **0.354** | **0.532** | **0.600** | **0.586** | **0.450** | **0.472** |
| MEX (n=83) | **0.489** | - | -0.007 | -0.006 | **0.286** | -0.003 | 0.003 | -0.001 | -0.021 | -0.004 | -0.010 | -0.010 | -0.006 |
| DAR (n=100) | **0.407** | -0.005 | - | -0.003 | **0.251** | -0.007 | -0.018 | -0.006 | -0.031 | 0.006 | -0.004 | -0.012 | -0.003 |
| WOL (n=31) | **0.607** | -0.010 | -0.001 | - | **0.256** | 0.012 | 0.018 | 0.012 | -0.019 | -0.020 | -0.028 | -0.018 | -0.010 |
| SGA (n=31) | **0.946** | **0.286** | **0.287** | **0.276** | - | **0.306** | **0.498** | **0.271** | **0.454** | **0.226** | **0.246** | **0.237** | **0.233** |
| NCR (n=64) | **0.426** | -0.005 | -0.011 | 0.004 | **0.327** | - | -0.027 | -0.009 | -0.029 | 0.016 | 0.010 | -0.006 | 0.005 |
| SCR (n=14) | **0.564** | -0.018 | -0.031 | -0.018 | **0.479** | -0.031 | - | -0.027 | -0.042 | 0.029 | 0.017 | -0.014 | 0.005 |
| PAN (n=136) | **0.365** | 0.001 | -0.006 | 0.012 | **0.299** | -0.010 | -0.029 | - | -0.027 | 0.019 | 0.010 | -0.003 | 0.006 |
| MAL (n=18) | **0.508** | -0.014 | -0.028 | -0.001 | **0.507** | -0.030 | -0.049 | -0.029 | - | 0.000 | -0.015 | -0.031 | -0.018 |
| TRI (n=36) | **0.615** | -0.003 | 0.010 | -0.026 | **0.248** | 0.012 | -0.007 | 0.023 | 0.016 | - | -0.023 | -0.008 | -0.012 |
| BVA (n=34) | **0.600** | -0.009 | 0.000 | -0.030 | **0.270** | 0.004 | -0.017 | 0.013 | 0.000 | -0.025 | - | -0.018 | -0.011 |
| SAN (n=47) | **0.504** | -0.009 | -0.009 | -0.020 | **0.276** | -0.007 | -0.031 | 0.000 | -0.018 | -0.012 | -0.019 | - | -0.009 |
| ECU (n=68) | **0.469** | -0.007 | -0.006 | -0.011 | **0.277** | -0.006 | -0.027 | 0.000 | -0.017 | -0.006 | -0.009 | -0.012 | - |

Subpopulation abbreviations and grouping of previously published data:

HAW, Hawai’i (Duncan et al. 2006)

MEX, Mexican Pacific (samples grouped from: Baja California, La Paz, and Mazatlan; Duncan et al. 2006; Nance et al. 2011)

DAR, Darwin Arch (this study)

WOL, Wolf Island (this study)

SGA, South Galápagos (this study)

NCR, North Costa Rica (samples grouped from: Tarcoles and Golfo de Nicoya; Nance et al. 2011; this study)

SCR, South Costa Rica (Golfo Dulce; this study)

PAN, Panama (samples grouped from: Panama location unknown, Golfo de Montijo, Chiriqui, and Bahia de Parita; Duncan et al.

2006; Nance et al. 2011; this study)

MAL, Malpelo Island (Quintanilla et al. 2012)

TRI, Utria-Tribuga (samples grouped from: Tribuga and Utria; Quintanilla et al. 2012; this study)

BVA, Buenaventura (Quintanilla et al. 2012; this study); SAN, Sanquianga (Quintanilla et al. 2012; this study)

ECU, Ecuador (samples grouped from: Cojimies, South Ecuador, and Manta; Quintanilla et al. 2012; this study).

Sample sizes appear in brackets next to sample location abbreviations.

Figure S1. POWSIM output (Proportion of Significance vs. Average *F*_ST_ across runs) used to assess the power of the nine microsatellite DNA marker set and the sample sizes used in the present study to detect genetic differentiation of scalloped hammerheads via Chi-square (blue square) and Fisher’s exact test (orange circle). An effective population size (N_e_) of 500 individuals was assumed for both datasets. A) POWSIM output for the global dataset (ETP, SEY and FLA), and B) POWSIM output for the ETP dataset.
Subpopulation abbreviations: ETP, Eastern Tropical Pacific; SEY, Seychelles; FLA, Florida, USA.


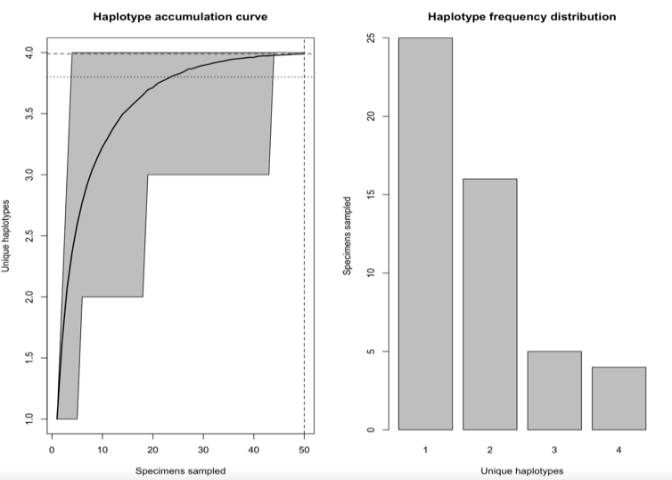

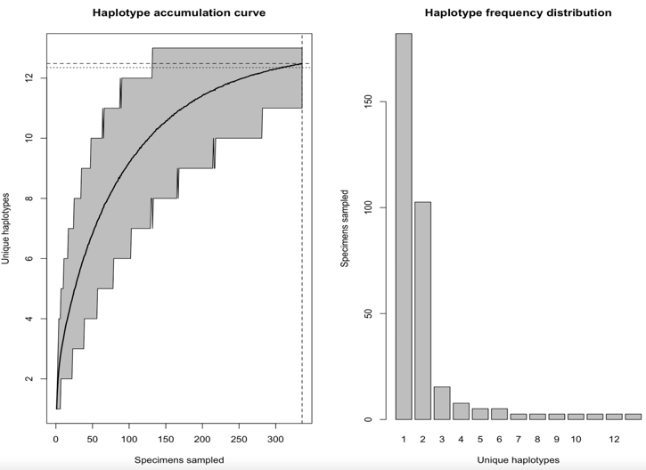


1. Galápagos aggregation adults
2. Seychelles adults
3. Florida adults


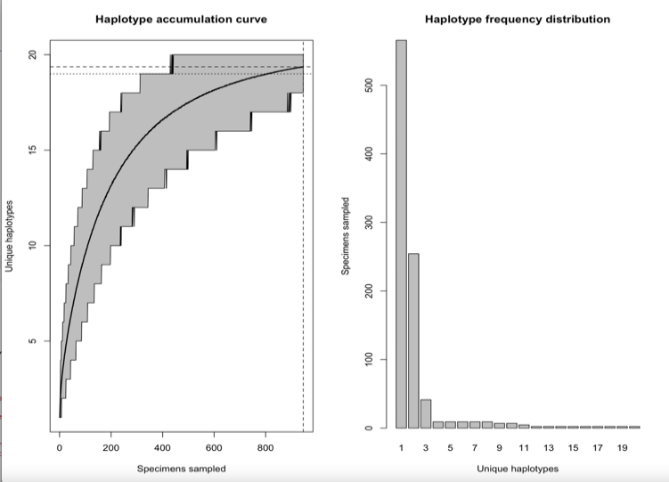


D. Eastern Tropical Pacific (adults and YOY)

Figure S2. Rarefaction curves (Unique haplotypes vs. specimens sampled) of mitochondrial control region haplotype accumulation across *Sphyrna lewini* subpopulations: A) Galápagos adult aggregation, B) Seychelles adults, C) Florida adults, and D) the overall Eastern Tropical Pacific (adults and YOY).
